# Optimization of biomass pretreatments using fractional factorial experimental design

**DOI:** 10.1186/s13068-018-1200-2

**Published:** 2018-07-24

**Authors:** Camila A. Rezende, Beatriz W. Atta, Marcia C. Breitkreitz, Rachael Simister, Leonardo D. Gomez, Simon J. McQueen-Mason

**Affiliations:** 1 Institute of Chemistry, University of Campinas-UNICAMP, P.O. Box 6154, Campinas, SP 13083-970 Brazil; 2 Centre for Novel Agricultural Products-CNAP, University of York, Heslington, York, YO10 5YW UK

**Keywords:** Design of experiment, DOE, Fractional experimental design, Pretreatment, Elephant grass, Ethanol, Biofuel

## Abstract

**Background:**

Pretreatments are one of the main bottlenecks for the lignocellulose conversion process and the search for cheaper and effective pretreatment methodologies for each biomass is a complex but fundamental task. Here, we used a 2ν^5−1^ fractional factorial design (FFD) to optimize five pretreatment variables: milling time, temperature, double treatment, chemical concentration, and pretreatment time in acid–alkali (EA) and acid–organosolv (EO) pretreatments, applied to elephant grass leaves.

**Results:**

FFD allowed optimization of the pretreatment conditions using a reduced number of experiments and allowed the identification of secondary interactions between the factors. FFD showed that the temperature can be kept at its lower level and that the first acid step can be eliminated in both pretreatments, without significant losses to enzymatic hydrolysis. EA resulted in the highest release of reducing sugars (maximum of 205 mg/g substrate in comparison to 152 mg/g in EO and 40 mg/g in the untreated sample), using the following conditions in the alkali step: [NaOH] = 4.5% w/v; 85 °C and 100 min after ball milling the sample. The factors statistically significant (*P *< 0.05) in EA pretreatment were NaOH concentration, which contributes to improved hydrolysis by lignin and silica removal, and the milling time, which has a mechanical effect. For EO samples, the statistically significant factors to improved hydrolysis were ethanol and catalyst concentrations, which are both correlated to higher cellulose amounts in the pretreated substrates. The catalyst is also correlated to lignin removal. The detailed characterization of the main hemicellulosic sugars in the solids after pretreatments revealed their distinct recalcitrance: glucose was typically more recalcitrant than xylose and arabinose, which could be almost completely removed under specific pretreatments. In EA samples, the removal of hemicellulose derivatives was very dependent on the acid step, especially arabinose removal.

**Conclusion:**

The results presented herewith contribute to the development of more efficient and viable pretreatments to produce cellulosic ethanol from grass biomasses, saving time, costs and energy. They also facilitate the design of enzymatic cocktails and a more appropriate use of the sugars contained in the pretreatment liquors, by establishing the key recalcitrant polymers in the solids resulting from each processing step.

**Electronic supplementary material:**

The online version of this article (10.1186/s13068-018-1200-2) contains supplementary material, which is available to authorized users.

## Background

Bioethanol is an alternative energy vector that can be obtained by direct fermentation of starch and sugar-based feedstocks, known as first-generation ethanol, or by hydrolysis of polymers contained in plant biomass, the so-called second generation or lignocellulosic ethanol. First-generation ethanol is a reality in countries like Brazil, where the total amount of ethanol produced in the 2015/2016 harvest reached 30 billion liters, of which 26 billion liters were consumed in the internal market, against 74 billion liters of gasoline [1]. Lignocellulosic ethanol represents a sustainable way of producing low carbon biofuels without negative consequences for food security and can also be beneficial to provide a profitable use of agricultural wastes, most of which are presently underutilized [2, 3]. However, commercial production has so far failed to cause a significant impact in the energy matrix due to high costs and the large volumes of feedstock required.

The efficient conversion of different biomass sources to bioethanol depends on pretreatment processes to decrease cell wall recalcitrance and to allow higher hydrolysis yields [4, 5]. Different pretreatments with variable costs and efficiencies have been tested in different biomasses, including milling and irradiation, hot water/steam explosion, ammonia fibre explosion, organic and ionic solvents, supercritical fluids, diluted acids and/or bases [2, 6–10]. Pretreatment methods alter the structure and the chemical composition of the lignocellulosic matrix in a number of different ways: by increasing the porosity and the surface area accessible to enzymes; by altering the hydrophilicity of the substrate; by removing hemicellulose and lignin; or by decreasing the cellulose degree of polymerization and crystallinity [4, 8, 11, 12]. An ideal pretreatment strategy should also be cost effective, thus minimizing the energy input, the operational time, and the amount of residuals produced. Finally, effective pretreatments should not lead to carbohydrate degradation and the production of enzyme inhibitors and toxic products for fermenting microorganisms [2, 4, 6].

Diversification of the possible biomass feedstocks for lignocellulosic derived fuels is important to meet the rising demand for bioethanol and also to replace the enormous amount of fossil fuels currently consumed. Moreover, the search for new biomass sources will help to assure the uninterrupted operation of ethanol plants throughout the year, unlike a seasonal operation in which the use of the invested capital is inefficient and workers are intermittently hired [13]. Non-food lignocellulosic biomasses, such as elephant grass, are a potential source of abundant and sustainable feedstock, not only for energy production but within a more comprehensive biorefinery approach. Elephant grass (*Pennisetum purpureum*) is a C4 plant, usually cultivated for cattle feed and that can be harvested up to four times a year. While sugarcane bagasse produces 21 ton of dry matter/ha/year (sugar and bagasse) and corn produces 10 ton/ha/year (grains and stover), elephant grass production can reach 45 ton of dry matter/ha/year [14]. Its high potential as a biomass source to produce second-generation ethanol has attracted attention and a number of studies have been carried out in this species [8, 13, 15–17].

Although there is consensus in the literature that pretreatments are essential to enable practical hydrolysis yields, the optimization of the most adequate pretreatment methods for a specific biomass type is a complicated task that will depend on the combination of intrinsic characteristics of the plant biomass (for instance, type, organ and age of the plant) and on the pretreatment conditions applied [2]. The stringency of a pretreatment is defined by a number of factors, the most relevant among them being the reactant concentration, temperature, time, pressure, solid to liquid ratio, and the presence of catalysts [18]. Therefore, the use of approaches involving design of experiments (DOE) is a valuable tool to optimize the experimental trials in these systems, thus allowing improved final responses to be obtained, with a reduced number of experiments [19].

DOE is a multivariate technique that has been largely used across many disciplines to extract meaningful information for the development of products, processes and methods. It examines the influence of different experimental factors simultaneously and the identification of interactions among them, which cannot be achieved by the traditional one-factor-at-a-time approach [20]. In a factorial design, a set of predefined experiments is determined to combine levels of the experimental (independent) variables and connect to the properties of interest (dependent variables) by models generated by multiple linear regression (MLR). These models allow the construction of response surface graphs to describe the behaviour of the system all over the experimental domain, and not only where experiments were performed. To preserve important information, a high-resolution FFD should be preferred, for example resolution V designs [19], where the main effects are aliased only with fourth order interactions, which tend to be not significant. Two-factor interactions will be aliased with three-factor interactions. This is, therefore, an excellent design to reduce the number of runs and still obtain accurate results.

Due to the potential of experimental design for the multifactorial study of variables in pretreatments, the number of publications using these methodologies has increased in the literature [5, 8, 21, 22]. For instance, Rabelo et al. used DOE (2^4^ full factorial) to evaluate the need for particle size reduction in sugarcane bagasse prior to an alkaline hydrogen peroxide pretreatment and also to optimize the pretreatment conditions and the enzyme loads in enzymatic hydrolysis [22]. In an acid-mediated steam explosion pretreatment applied to elephant grass, the influence of acid concentration, reaction time and temperature was also evaluated using a 2^3^ central composite design with three levels to each variable [8]. In the present work, a 2ν^5−1^ fractional factorial design was applied to study the effect of five independent variables in two different pretreatment methodologies (acid–alkali and acid–organosolv) applied to elephant grass leaves. This approach allowed optimizing the experimental conditions of each pretreatment with fewer experiments than in a full factorial design. The correlations found between the chemical composition changes and the saccharification results contributed to the understanding of mechanisms involved in the increase of digestibility in the substrates.

## Results and discussion

### Effect of pretreatments on saccharification

**Fig. 1:**
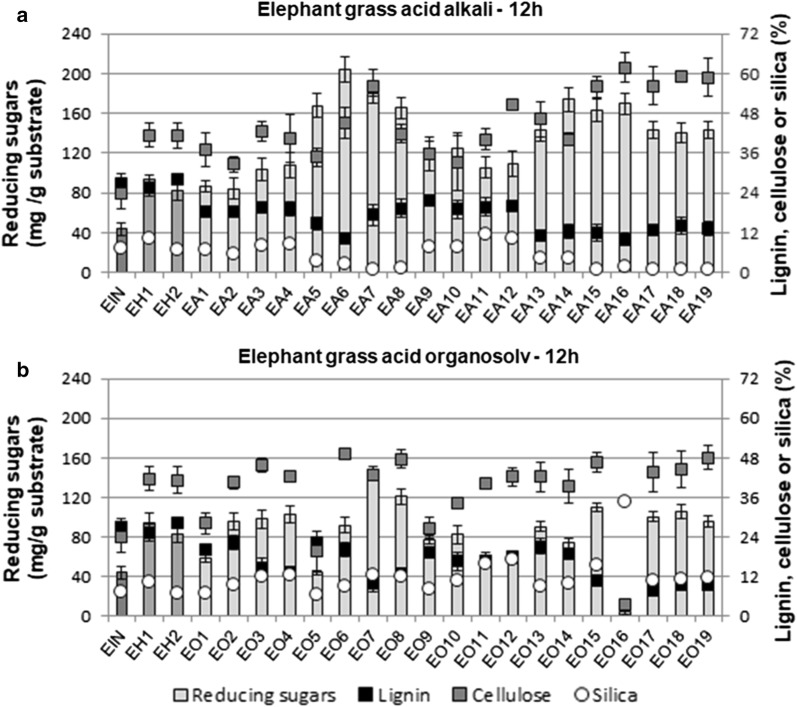
Reducing sugars released from substrates before and after 12 h enzymatic hydrolysis and their percentages of acetyl bromide soluble lignin, crystalline cellulose and silica: **a** elephant grass pretreated with acid–alkali (EA) and **b** with acid–organosolv (EO). Reducing sugars (mg/g substrate) are indicated by grey bars in the left axis, while lignin (black squares), cellulose (grey squares) and silica (white circles) are indicated in the right axis. Error bars are standard deviation values from replicates

Figure 1 shows the reducing sugars obtained after enzymatic saccharification of elephant grass leaves after 12 h of hydrolysis at 50 °C, together with lignin, cellulose and silica percentages in the solids after pretreatments. Sample names are specified in Tables 2 and 3, according to the pretreatment conditions. In general, the samples pretreated under the acid–alkali methodology (Fig. 1a) presented higher sugar release than the ones pretreated using the acid–organosolv method (EO-Fig. 1b), thus revealing a distinct response of this biomass to different pretreatments.

All the EA samples released more reducing sugars than elephant grass in natura (EIN). While EIN resulted in ca. 40 mg of sugar/g substrate, EA-pretreated samples reached up to 200 mg/g substrate (EA6 in Fig. 1a). Samples that underwent only the acid step, using 1% H_2_SO_4_ (EH1) or 2% H_2_SO_4_ (EH2), also showed improved sugar release that was twice as high as in the untreated samples (EIN). The majority of the EO samples also showed improved sugar release when compared to EIN (Fig. 1b), reaching up to 144 mg/g in EO7. Only two pretreatment conditions showed no saccharification improvements: samples EO5 and EO16 (Fig. 1b). EO5, where a very mild pretreatment was applied (no acid; no catalyst; 80% ethanol; 160 °C; 30 min), can be expected to have a low performance. EO16 was pretreated using all the experimental conditions at high levels (1% H_2_SO_4_; 0.06 M catalyst; 80% ethanol; 200 °C; 90 min, as shown in Table 3), and the solids were carbonized, indicating that the conditions were too severe, thus producing degradation of sugars. Soluble acetyl bromide lignin of sample EO16 could not be determined due to that high background.

For EA samples, the most promising conditions in terms of sugar release are the ones where NaOH was used at higher concentrations (E5–E8 and E13–E16 in Fig. 1a). NaOH removes lignin and all the pretreatments including the alkali step (samples EA1–EA19) resulted in lower lignin contents in comparison to samples where the alkali step was not applied (EIN, EH1 and EH2). While EIN presents lignin content around 27% w/w, this percentage can decrease to near 10% under certain pretreatment conditions. A negative correlation is observed between high sugar release and low lignin levels for most of EA samples (EA samples (*R* = − 0.67, Additional file 1: Figure S1)), which conforms with previously published results where hydrolysis yields increase as the lignin levels decrease due to alkali hydrolysis [7, 13, 23]. Although the correlation presented here does not necessarily confirm a causal relationship, it can be useful to identify meaningful correlations.

In EO samples, the correlation coefficient of reducing sugars as a function of the lignin content is − 0.67 (Additional file 1: Figure S4), similar to EA samples. Increased sugar release in EO can also be correlated to low lignin levels in many samples (EO3, EO4, EO7, EO8, EO15, EO17, EO18, and EO19 in Fig. 1b), and low release of sugars can be associated with higher lignin contents (EIN, EO1, EO5, EO11, EO12, EO14). Despite this, some discrepancies can also be observed, such as in the pairs EO1 and EO2 and EO5 and EO6, which have similar lignin levels, but very different results for sugar release.

In terms of the cellulose content, higher sugar release correlates with higher cellulose contents only in some of the EA samples in Fig. 1a (*R* = 0.41, Additional file 1: Figure S2). On the contrary, in EO samples, sugar release is strongly correlated to the cellulose content (*R* = 0.80, Additional file 1: Figure S5). These results point out to a dependence of hydrolysis efficiency with compositional factors other than the cellulose content, and also with factors that may not be related to composition but to morphology, such as substrate porosity and the distribution of the components.

Saccharification also shows a degree of correlation with the silica amounts in EA samples (*R* = − 0.71, Additional file 1: Figure S3). Silica is an important component in elephant grass biomass and its contents varied between 0.7 to 12% in EA samples (Fig. 1a), and from 7 to 17% in EO (Fig. 1b). It also represents the main inorganic fraction in this biomass, as observed by the comparison between the silica and the total ash amounts in Additional file 1: Figure S7. Grasses are generally rich in silica (SiO_2_), which is taken up from the soil in the form of silicic acid (Si(OH)_4_) and deposited as incrustations of amorphous silica inside the plant cell walls or in intercellular spaces [13, 24].

In Fig. 1a, higher saccharification yields were observed in EA samples with lower silica contents (EA5–EA8 and EA13–EA19), which also correspond to samples pretreated under high or medium NaOH concentrations (Table 2). This indicates the removal of not only lignin but also silica by NaOH from elephant grass samples.

Silica solubilization, which depends on its depolymerization into silicic acid (Eq. 1), is a reaction catalysed both in alkaline and acid pH, but it is more favourable in alkali medium (pH > 9), where the adsorption and repolymerization of silicic acid are unlikely to occur.

1 $${\text{SiO}}_{{ 2({\text{s}})}} \, + \, 2 {\text{ H}}_{ 2} {\text{O}}_{{({\text{l}})}} \, \rightleftarrows \,{\text{Si}}\left( {\text{OH}} \right)_{{ 4({\text{aq}})}}$$

Effective pretreatments to remove silica from biomasses are fundamental to allow their use for cellulosic ethanol production, since silica acts as a physical barrier hindering enzymatic degradation [25]. Besides this, silica is a problem in industrial processes because it forms water-insoluble precipitates that block filtration systems and damage equipment. Despite this, silica is a valuable by-product that can be extracted for use in other relevant applications, such as the production of catalysts and of mesoporous structured silica for adsorption processes [25].

In the case of EO samples, very low correlation (*R* = 0.20) is observed between the sugar release and the silica content (Additional file 1: Figure S6). It can be observed in Fig. 1b that silica is not properly removed by the acid–organosolv pretreatment, since the silica percentages are typically high in all the samples. Sample EO16 had exceptionally high silica content (35%), probably due to degradation of the other less recalcitrant components of the elephant grass during pretreatments, as previously discussed.

Silica solubilization could occur in the organosolv pretreatment via the acid-catalysed mechanism of the reaction in Eq. 1, since this pretreatment medium contains 0.06 M of acid catalyst in some cases and is not anhydrous, which are both required conditions for silica solubilization [25]. Conversely, silica could be efficiently removed from elephant grass by the alkali pretreatment applied here, using NaOH concentrations equal to or higher than 2.5% w/v. According to the results in silica quantification, the alkali route is much more effective for silica removal than the acid one.

### Pretreatment effects in the removal of hemicellulose sugars from the samples

**Fig. 2:**
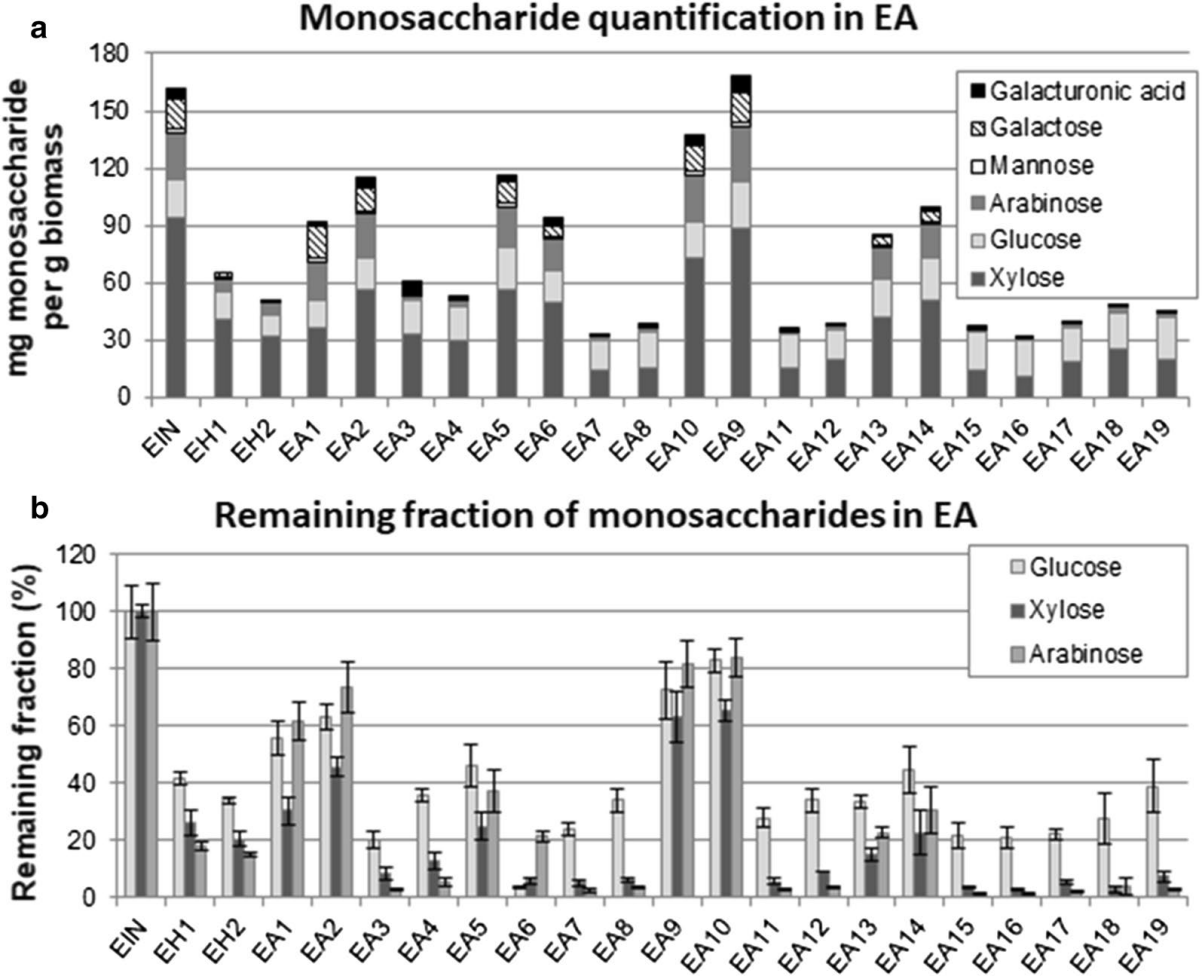
**a** Quantification of hemicellulose monosaccharides (in mg/g substrate) and **b** percentages of glucose, xylose and arabinose remaining in the solid substrates of elephant grass before and after pretreatments with acid–alkali (EA)

**Fig. 3:**
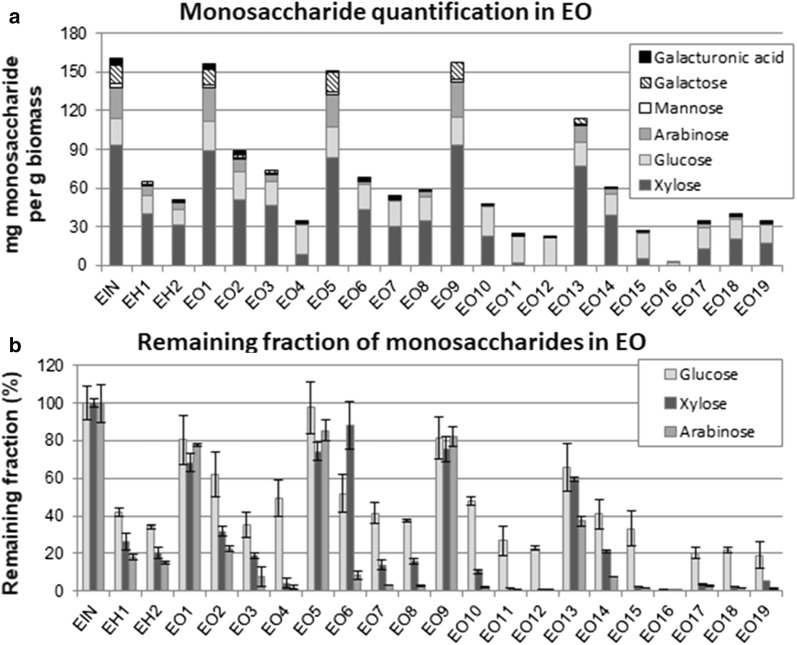
**a** Quantification of hemicellulose monosaccharides (in mg/g substrate) and **b** percentages of glucose, xylose and arabinose remaining in the solid substrates of elephant grass before and after pretreatments with acid–organosolv (EO)

Detailed composition of the sugars derived from the hemicellulose fraction for EA and EO samples as well as the total hemicellulose amount is shown in Figs. 2a and 3a, respectively. The maximum amounts of hemicellulosic sugars quantified in these samples were ca. 160 mg/g (EIN, EA9 in Fig. 2a, and EO1, EO5 and EO9 in Fig. 3a) and the main monosaccharides are xylose, glucose and arabinose. Figure 2a shows that the total amount of hemicellulose in EA samples decreases more sharply with the pretreatments that include the acid step (EH1–2, EA3–4, EA7–8, EA11–12, EA15–19), and that the use of a 2% v/v H_2_SO_4_ concentration (EH2) removes a slightly larger amount of hemicellulose than a 1% v/v H_2_SO_4_ solution (EH1). Also, the total hemicellulose (sum of the monosaccharide fractions) in samples that underwent both the acid and the alkali step (EA3–4, EA7–8, EA11–12, EA15–19) is not very different from the hemicellulose amount in samples that underwent the acid step only (EH1–2). This is in agreement with previous observations in the literature, where acid hydrolysis is mainly responsible for hemicellulose extraction [5, 7, 13].

In EO samples (Fig. 3a), the acid step is also important to hemicellulose removal, but other factors such as the presence of the catalyst (0.06 M H_2_SO_4_) and temperature in organosolv step and the interaction between the two first effects are also important. Hemicellulose content in samples EO1, EO5, EO9 and EO13 (to a certain extent) is very similar to EIN. In these samples, the pretreatment was carried out without the first acid step and no catalyst was used in the organosolv step. These results indicate the hydrolytic role of the acid used in the organosolv step in removing hemicellulose. Figure 3a also shows that the hemicellulose amounts tend to decrease in the groups of samples from EO1–EO4, EO5–EO8, EO9–EO12, and EO13–EO16, according to the severity of the pretreatments.

The different monomeric sugars present in the hemicellulose fractions in elephant grass samples indicate different recalcitrance to hydrolysis under different pretreatment conditions. Figures 2b and 3b show the remaining fractions of the main hemicellulose constituents (glucose, xylose and arabinose) in EA, and EO, respectively, as determined in the solids after the different pretreatment conditions. The remaining fraction of each of these monosaccharides was obtained considering their content in the solid samples after pretreatment and also the pretreatment yield (weight of the remaining dried solid after the pretreatment as compared to the solid weight of untreated biomass).

Figure 2b shows that the xylose remaining fraction in EA is lower than the glucose fraction in all the samples, except in EA6 and EA13, where the amounts are similar for both monosaccharides. This indicates that the xylose fraction is more likely to be removed from the biomass by the acid–alkali pretreatment than glucose. The exceptions to this (EA6 and EA13) are pretreatments with high NaOH load (4.5% m/v) and long reaction time (100 min), conditions severe enough to remove both monomers equally. The arabinose content in EA presents an interesting dynamic depending on the acid step. The remaining arabinose fraction follows the same pattern of xylose in all the samples where an acid step was applied, being more easily removed than glucose and showing a final remaining amount close to xylose values. In samples in which the acid step was not carried out (EA1–2, EA5–66, EA9–10, EA13–14), arabinose presents remaining values higher than xylose and closer to glucose.

In EO samples (Fig. 3b), two main profiles are observed in terms of removal of hemicellulose fractions after pretreatments. The most frequent is glucose being more recalcitrant than xylose and arabinose, as observed in samples EH1, EH2, EO2–EO4, EO7, EO8, EO10–EO12, EO 15, EO17–EO19. The second profile is observed in samples EO1, EO5 and EO9, where the hemicellulose content is poorly removed as these three experimental conditions are relatively mild.

In summary, these results show a strong influence of pretreatment conditions in the final composition of this biomass in terms of the total hemicellulose amount but also of its fractions. Monosaccharides, such as xylose and arabinose, which presented initial percentages very similar to glucose in EIN are almost completely removed under specific pretreatment conditions and should not represent a problem for cellulose hydrolysis. This should influence the choice of enzymatic cocktails to hydrolyse these samples more efficiently.

### Experimental design

#### Elephant grass pretreated with acid–alkali (EA)

**Fig. 4:**
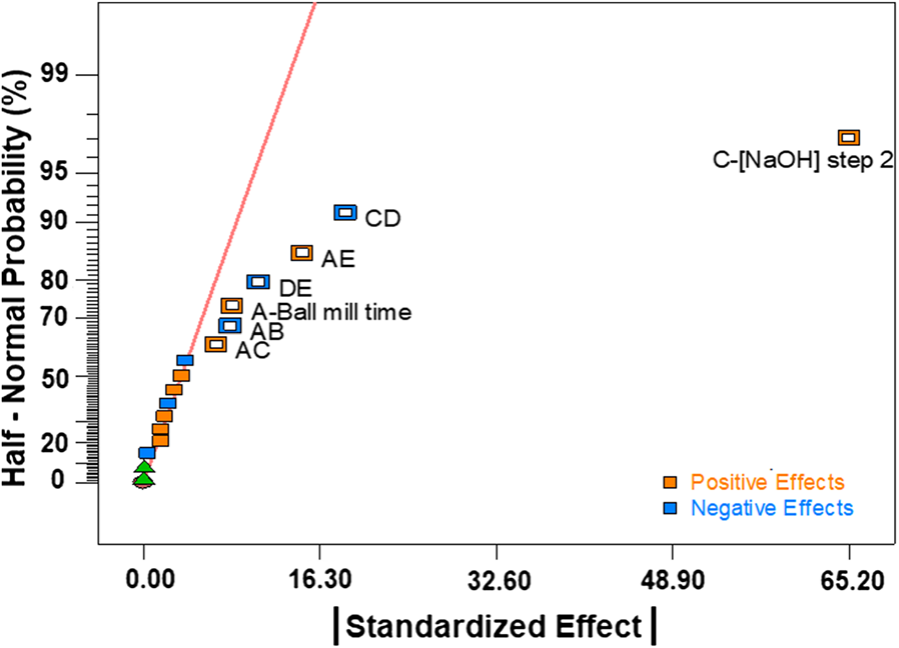
Half-normal plot of the standardized effects (effects/errors) for elephant grass samples pretreated with acid–alkali (EA)

Figure 4 shows the factors influencing the sugar release in EA samples, where the significant effects are those that deviate from the straight line centered in zero. NaOH concentration in step 2 has the highest effect on sugar release, while ball milling time has a minor but still significant effect. Both are positive, indicating a direct correlation with the release of reducing sugars. There are several significant binary interactions ([NaOH] × Temperature (CD); Ball Mill × Time (AE); Temperature × Time (DE); Ball mill × [H_2_SO_4_] (AB); Ball Mill × [NaOH] (AC)), which highlight the importance of the use of the DOE approach to study this system. The analysis of variance of the model containing the significant coefficients (in addition to B, D and E that were added to keep a hierarchical order in the model) is presented in Table 1.

**Table 1:** ANOVA table of the model describing the sugar release as a linear function of the selected coefficients for EA samples

Source	Sum of squares	Degrees of freedom	Mean square	*F* value	*P* value
Regression	20,497	10	2049.70	87.95	4.49E−07
A-ball mill time	270.6025	1	270.60	11.61	9.26E−03
B-[H_2_SO_4_] step 1	0.7225	1	0.72	0.03	8.65E−01
C-[NaOH] step 2	17,004.16	1	17,004.16	729.64	3.80E−09
D-temperature step 2	11.2225	1	11.22	0.48	5.07E−01
E-time step 2	50.41	1	50.41	2.16	1.80E−01
AB	260.8225	1	260.82	11.19	1.01E−02
AC	182.25	1	182.25	7.82	2.33E−02
AE	864.36	1	864.36	37.09	2.93E−04
CD	1398.76	1	1398.76	60.02	5.50E−05
DE	453.69	1	453.69	19.47	2.25E−03
Residual	186.437895	8	23.30		
Lack of fit	181.017895	6	30.17	11.13	8.47E−02
Pure error	5.42	2	2.71		
Cor total	20,683.4379	18			

The calculated *F* value considering the regression mean square and the residual mean square (MS_REG_/MS_RES_) is equal to 87.95, which is much higher than the tabulated *F* value (10, 8, 95% confidence level) of 3.35, indicating that the regression is highly significant. The calculated *F* value considering the lack of fit mean square and the pure error mean square (MS_LOF_/MS_PE_) is 11.13, lower than the tabulated *F* value (6, 2, 95% confidence level) of 19.30, indicating fit of the linear model.

**Fig. 5:**
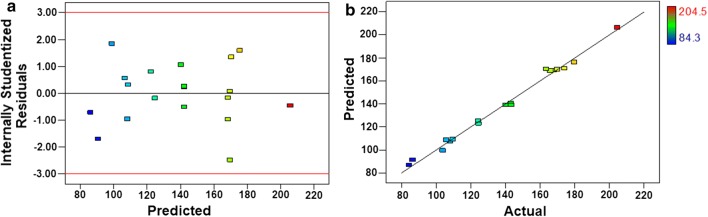
**a** Internally studentized residuals (residuals/standard deviation of the regression) vs predicted values of reducing sugars as provided by the selected model obtained for EA samples and **b** predicted values vs actual experimental values of reducing sugars

The diagnostics graphs of residuals vs predicted values for EA samples (Fig. 5a) indicate a random distribution of the residuals without heteroscedasticity and no outliers were observed. The graph of predicted vs actual experimental responses (Fig. 5b) shows that the data fit the linear model for EA pretreatments as predicted.

**Fig. 6:**
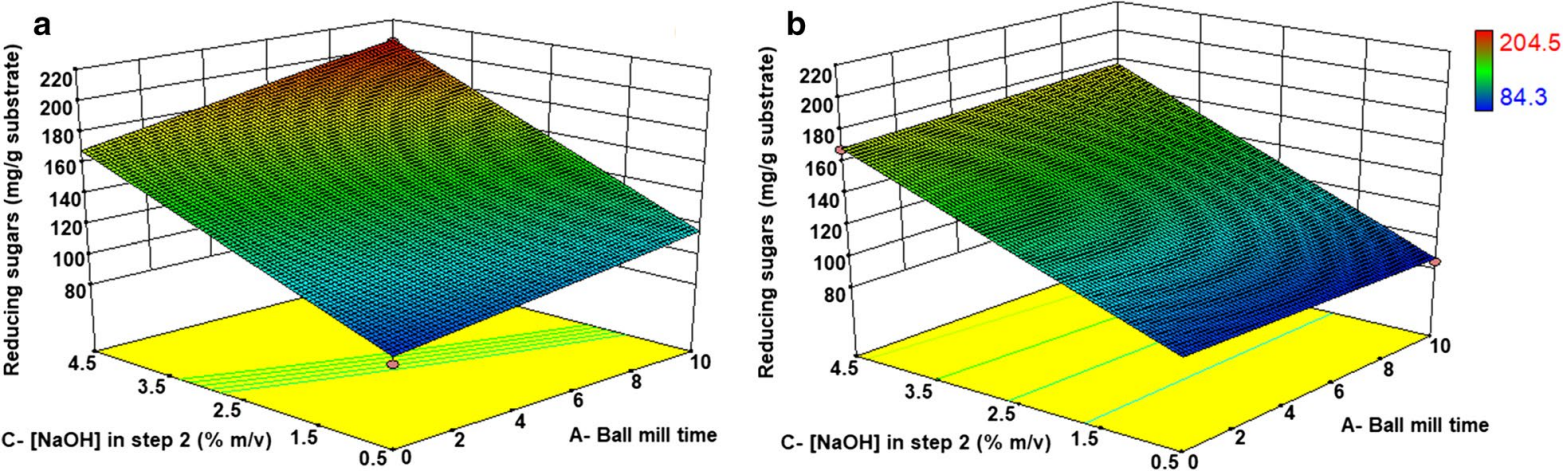
Response surfaces indicating the characteristics of the reducing sugar release as a function of the two most important factors for EA samples: NaOH concentration and ball mill time. The other factors were kept constant in **a** [H_2_SO_4_] in step 1 = 0%; time = 100 min and temperature of step 2 = 85 °C, and in **b** [H_2_SO_4_] in step 1 = 0%; time = 20 min and temperature of step 2 = 85 °C

The response surface is shown in Fig. 6a, where the highest values of reducing sugars released can be obtained using the higher concentration of NaOH (4.5% m/v) and a 10 h ball milling time. The interaction between the two factors is observed comparing the two edges of the surface that are not parallel, i.e., the ball mill time influences the sugar release in a more pronounced way when NaOH is increased. The shape of the response surface suggests that a displacement towards higher concentrations of NaOH and ball milling time could provide even higher values of sugar release, probably leading to a maximum, where a quadratic model could be adjusted. Within this experimental domain, the highest predicted value of sugar release is 205.2 mg/g substrate, achieved using the following conditions: [NaOH] = 4.5%, ball mill time = 10 h, [H_2_SO_4_] in step 1 = 0%; Temperature = 85 °C and Time of step 2 = 100 min, which correspond to the condition EA6 in Table 2 (experimental value = 204.5 ± 10.9 mg/g substrate of sugar release).

**Table 2:** Levels of the factors evaluated in the $2_{\text{V}}^{{ 5 { - 1}}}$ fractional factorial design, sample identification with the corresponding experimental conditions and the main response evaluated in the acid–alkali pretreatment applied to elephant grass leaves (EA)

	Low level (−)	High level (+)	Central (0)
Factor levels			
A-ball mill time (h)	0	10	5
B-[H_2_SO_4_] (%v/v)	None	2	1
C-[NaOH] (%w/v)	0.5	4.5	2.5
D-temperature (°C)	85	125	105
E-time (min)	20	80	60

Samples and experimental conditions						Response
Sample name	Ball mill time (h)	[H_2_SO_4_] step 1 (% v/v)	[NaOH] step 2 (% m/v)	Temp step 2 (°C)	Time step 2 (min)	Reducing sugar release (mg/g)
EA1	0	None	0.5	85	100	86.4
EA2	10	None	0.5	85	20	84.3
EA3	0	2	0.5	85	20	103.7
EA4	10	2	0.5	85	100	108.0
EA5	0	None	4.5	85	20	168.0
EA6	10	None	4.5	85	100	204.5
EA7	0	2	4.5	85	100	179.7
EA8	10	2	4.5	85	20	165.8
EA9	0	None	0.5	125	20	124.4
EA10	10	None	0.5	125	100	124.3
EA11	0	2	0.5	125	100	105.7
EA12	10	2	0.5	125	20	109.5
EA13	0	None	4.5	125	100	143.0
EA14	10	None	4.5	125	20	173.9
EA15	0	2	4.5	125	20	163.3
EA16	10	2	4.5	125	100	169.7
EA17	5	1	2.5	105	60	143.1
EA18	5	1	2.5	105	60	140.3
EA19	5	1	2.5	105	60	143.2

Temperature does not have a significant effect by itself, but its interaction with NaOH concentration (CD) would have a negative effect on sugar release (Fig. 4). For this reason, an additional experiment was carried out under very similar conditions to EA6, except that the temperature was kept at 125 °C, obtaining a total of 192.2 ± 4.9 mg/g substrate of sugars. This result does not show a significant change in sugar release within this temperature range, indicating that the temperature can be kept at 85 °C to obtain the same amount of sugars with a reduced energy input.

The reaction time is also an individually insignificant variable, but it influences the interaction with ball milling time. Indeed, a change in sugar release is observed when the time is shortened (predicted value = 177.4 mg/g, Fig. 6b), thus showing that this variable is more favourable at its highest level (100 min).

The release of reducing sugars can be optimized in elephant grass samples pretreated by the alkali method by maximizing the NaOH concentration (4.5% m/v), even if the temperature is kept at its low level (85 °C) to reduce processing costs. The reaction time, on the other hand, produces a maximum effect at the longer retention time (100 min). The first acid step can be eliminated since it did not contribute to improving hydrolysis in the case of these samples. Otherwise, in previous studies, acid–alkali pretreatment applied in two steps to sugarcane bagasse resulted in better hydrolysis efficiency than the alkali pretreatment alone [7]. The elimination of this step could contribute to reduce the pretreatment costs and time, and the volume of residues produced.

Finally, the use of a first step of mechanical pretreatment in a ball milling showed an important contribution to improve sugar release. Besides reducing the particle size, the main effect of ball milling is to reduce the sample crystallinity. In our ball mill, the maximum decrease in crystallinity that could be achieved was from 59% (sample in natura) to 50% after 10 h milling (Additional file 1: Figure S8), but more efficient mills should be able to provide similar or larger decreases in crystallinity in reduced times.

Along with sugar release, the main dependent variable measured in the experimental design, other variables related to saccharification can also be evaluated, such as the lignin and the silica contents. As previously discussed, the amount of sugar released in hydrolysis has a moderate correlation with both lignin (*R* = − 0.67 in Additional file 1: Figure S1) and silica (*R* = − 0.71 in Additional file 1: Figure S3). By calculating the values of the effects for this experimental design using lignin amount as a response, the only factor that seems to be relevant is the NaOH concentration and its interaction with the temperature (CD, Additional file 1: Table S2). In terms of silica, the relevant factors are NaOH concentration and its interaction with H_2_SO_4_ concentration (BC) (Additional file 1: Table S3). NaOH effect in these two responses is expected since lignin and silica removal from biomass by alkaline hydrolysis is a well-known fact [7, 25]. These results are also in accordance with the moderate but not insignificant correlation between these responses and points to an interesting dynamic of the evaluated factors towards the hydrolysis efficiency. While sugar release is directly influenced by ball milling time and alkali concentration, the latter contributes to improved sugar release by lignin and silica removal, whereas ball milling modifies sample morphology only.

#### Elephant grass pretreated with organosolv (EO)

**Fig. 7:**
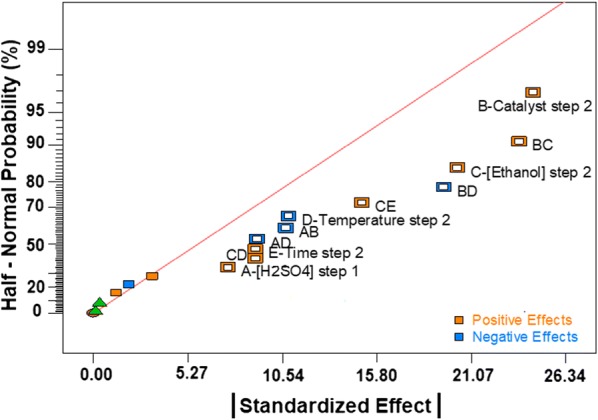
Half-normal plot of the standardized effects (effects/errors) for elephant grass samples pretreated with acid–organosolv (EO)

The half-normal plot of the effects on reducing sugar release for EO samples is shown in Fig. 7. The catalyst and the ethanol concentration in step 2 have the most significant effect. Both are positive, indicating that their increase improves the release of sugars. There are also interactions of the following factors: Catalyst × Ethanol (BC); Catalyst × Temperature (BD) and Ethanol × Time (CE). The coefficients D and E were included for hierarchical reasons. Furthermore, the coefficients A, AD, AB, CD were included because they improved the predictive capacity of the model due to the aliasing with significant effects (for example: AD = AD + BCE).

Similarly to sample EA, the diagnostics graphs of residuals vs predicted values (Additional file 1: Figure S10a) indicated a random distribution of the residuals without heteroscedasticity and the graph of predicted vs actual experimental responses (Additional file 1: Figure S10b) showed that the linear model describes well the experimental data. ANOVA indicated that the calculated *F* value of MS_REG_/MS_RES_ is equal to 11.17, which is higher than the tabulated *F* value (11, 4, 95% confidence level) of 5.9, thus indicating that the regression is significant. The calculated *F* value of MS_LOF_/MS_PE_ is 3.96, thus lower than the tabulated *F* value (4, 2, 95% confidence level) of 19.25, indicating no lack of fit of the linear model.

**Fig. 8:**
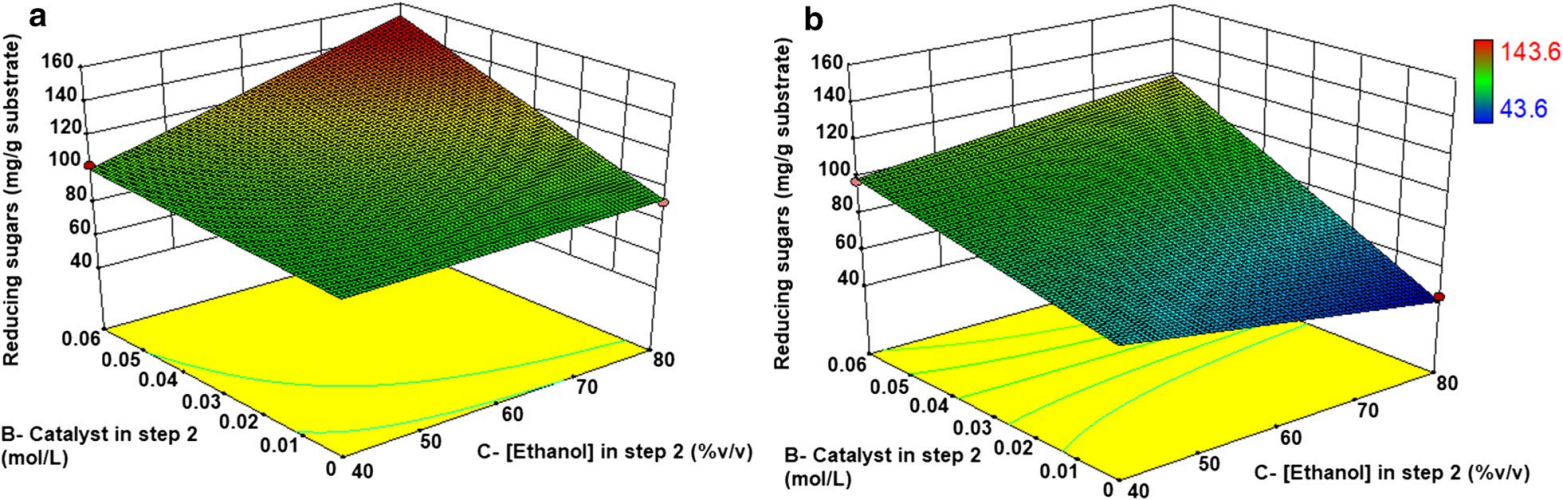
Response surfaces indicating the characteristics of the sugar release as a function of the two most important factors for EO samples: ethanol and catalyst concentrations, both in step 2. The other factors were kept constant in **a** [H_2_SO_4_] in step 1 = 1%; temperature of step 2 = 160 °C and time of step 2 = 90 min, and in **b** [H_2_SO_4_] in step 1 = 0% v/v; temperature of step 2 = 160 °C and time = 60 min)

The interaction between the catalyst and the ethanol concentration is shown in Fig. 8a. The increase in ethanol concentration caused an increase in the response when the catalyst concentration was at the highest level (0.06 mol/L). In the absence of catalyst, ethanol did not influence the response in a significant way.

The shape of the response surface indicates that a displacement towards higher values of ethanol and catalyst could increase sugar release. Inside this experimental domain, the highest predicted value of sugar release was 152.0 mg/g, which would be achieved using the following conditions: [Ethanol] in step 2 = 80% v/v; [catalyst] = 0.06 mol/L; [H_2_SO_4_] in step 1 = 1% v/v; Temperature in step 2 = 160 °C and Time in step 2 = 90 min. This value is slightly higher than the experimental value obtained with sample EO7 (143 ± 7.1 mg/g), in which all the conditions are identical to the predicted, except for the acid step. This indicates that the gain achieved by the acid step is not significant. Furthermore, maintaining the conditions used in EO7, but decreasing the reaction time to 60 min, the predicted sugar release decreases to 119.7 mg/g (using [Ethanol] = 80% v/v; [catalyst] = 0.06 mol/L; [H_2_SO_4_] = 0% v/v; Temperature = 160 °C and Time = 60 min), as shown in the surface response in Fig. 8b, indicating that a 90 min reaction time is more adequate. Finally, it is also important to highlight that the total amount of reducing sugars obtained by the organosolv method is lower than in the acid–alkali procedure.

Sugar release in EO presents a moderate negative correlation with lignin amount in these samples (*R* = − 0.67 in Additional file 1: Figure S4) and a positive higher correlation with the cellulose content (*R* = 0.80 in Additional file 1: Figure S5). These two responses were also evaluated in the experimental design and showed that the catalyst concentration is the only factor important for lignin removal, in agreement with their moderate correlation (Additional file 1: Table S5). Ethanol concentration is not important for lignin removal. Besides, while the catalyst effect is positive for sugar release (Fig. 7) it is negative for lignin removal (Additional file 1: Table S5), showing higher lignin removal when higher catalyst concentrations are used, in agreement with the negative correlation between sugar release and lignin amount. For cellulose amount, the significant factors are the concentrations of H_2_SO_4_ in step 1, catalyst and ethanol in step 2, and also the interaction between ethanol concentration and time (CE, Additional file 1: Table S6). The relatively strong correlation between sugar release and cellulose content in these samples can be explained by the three common influencing factors between them [catalyst and ethanol concentration and their interaction (CE)].

## Conclusions

Design of experiments (DOE) allowed simultaneous evaluation of several variables in acid–alkali and acid–organosolv pretreatments for elephant grass, a promising feedstock for lignocellulosic ethanol production. DOE allowed us to optimize pretreatments for this biomass excluding unnecessary steps and establishing more economical process conditions using lower temperatures and shorter pretreatment times. Alkali pretreatment preceded by ball milling was the most appropriate pretreatment for elephant grass compared to the acid–organosolv method, yielding a sugar release five times higher than the untreated sample. The detailed analysis of the hemicellulose fractions remaining in the solids after each of the different pretreatment conditions facilitates the planning of more adequate enzymatic cocktails to hydrolyse the solids. This detailed study of the main components being removed by pretreatments facilitates the assignment of the reaction liquors to future processing steps aiming at specific applications within a biorefinery concept.

### Experimental procedures

#### Biomasses and materials

Elephant grass leaves were kindly provided by Instituto de Zootecnia (Nova Odessa-SP, Brazil) from 10-month-old plants. Plant leaves were separated from the stalk and dried in a convection oven (Tecnal TE-394/3, Brazil) at 60 °C for 8 h, then knife milled (SOLAB—SL 31) until passing through a 2 mm sieve and stored in plastic boxes.

### Pretreatments and experimental design

#### Acid–alkali pretreatment

Elephant grass samples were pretreated using a sequential acid–alkali approach, which was based on the previous work with sugarcane bagasse [7]. It consists of a first step with diluted sulfuric acid (concentration lower than 2% v/v), followed by a second alkali step with NaOH solution (concentrations lower than 5% w/v). Pretreatment conditions were determined following a $2_{\text{V}}^{{ 5 { - 1}}}$ fractional factorial design, with triplicates in the central point to evaluate reducing sugars (mainly glucose) released in hydrolysis (dependent variable), as the main response. Lignin and silica percentages in the solid samples were also evaluated as secondary responses and are presented in Additional file 1: Table S1.

The five independent factors evaluated in the acid–alkali pretreatment were: (1) time of ball milling (varied from 0 to 10 h); (2) H_2_SO_4_ concentration in step 1 (no acid step to 2% v/v); (3) NaOH concentration in step 2 (varied from 0.5 to 4.5% w/v); (4) temperature in step 2 (from 85 to 125 °C) and (5) reaction time in step 2 (20–100 min). Table 2 shows the levels within which the effects are varied and the corresponding sample name.

Ball milling was conducted using a 10 L capacity mill at 30 rpm for 0 (no milling), 5 or 10 h (Table 2). A dried and previously knife milled sample (80 g) was sealed in a 10 L ceramic jar internally coated with zirconium oxide, together with zirconium oxide spheres (50 spheres of 1 cm radius and 50 spheres of 0.5 cm radius, according to the optimized conditions used in a previous study [11]). Milled samples were collected every 2 h to be characterized in terms of crystallinity by X-ray diffraction (XRD), as described in Additional file 1 (Crystallinity index section).

In the acid step, milled samples (only knife milled or also ball milled) were treated with aqueous H_2_SO_4_ at concentrations of 0.5, 1, and 2% (v/v), using a 1:10 (g/mL) solid to solution ratio. Moisture in the samples was considered to calculate this ratio. Samples indicated by “none” in the acid column in Table 2 were not submitted to this step. Pretreatment was carried out in an autoclave (Phoenix AV-75, Araraquara-SP, Brazil), according to the following temperature ramp: 15 min to reach 120 °C, then 40 min at 120 °C and 80 min to cool to room temperature. Pretreated solids were then separated by filtering in cotton tissue (150 thread count), rinsed with tap water until neutral pH and oven dried at 60 °C for 7 h before the alkali step. In the following step, samples were treated with aqueous NaOH solutions at 0.5, 2.5 and 4.5% (m/v), using a 1:10 (g/mL) solid to solution ratio, for 20, 60 or 80 min at 85, 105 or 125 °C (Table 2). Fifteen min to reach the pretreatment temperature and the 80 min to cool to room temperature were also applied here, but the pretreatment time indicated in Table 2 is the time at the constant pretreatment temperature (85, 105, or 125 °C). At the end of this step, solid samples were filtered, rinsed and dried as previously described.

#### Acid–organosolv pretreatment

Elephant grass samples were also pretreated using a sequential acid–organosolv method that consisted of a first diluted acid step, followed by a treatment with ethanol in water as a solvent. Five pretreatment factors were also evaluated in this sample, using a $2_{\text{V}}^{{ 5 { - 1}}}$ fractional factorial design with triplicate in the central point: (1) H_2_SO_4_ concentration in step 1 (no acid step to 1% v/v H_2_SO_4_ in water); (2) concentration of H_2_SO_4_ used as a catalyst in step 2 (varied from 0 to 0.06 mol/L); (3) ethanol concentration in step 2 (from 40% v/v in water to 80% v/v), (4) temperature in step 2 (from 160 to 200 °C) and (5) reaction time in step 2 (30–90 min). Table 3 shows the levels of the factors and the main response (reducing sugars released by enzymatic hydrolysis). Lignin and cellulose percentages in the solid samples were also evaluated as secondary responses and are presented in Additional file 1: Table S4.

**Table 3:** Levels of the factors evaluated in the $2_{\text{V}}^{{ 5 { - 1}}}$ fractional factorial design, sample identification with the corresponding experimental conditions and the main response evaluated in the organosolv pretreatment applied to elephant grass leaves (EO)

	Low level (−)	High level (+)	Central (0)
Factor levels			
A-[H_2_SO_4_] (%v/v)	None	1	0.5
B-[catalyst] (mol/L)	0	0.06	0.03
C-[ethanol] (%v/v)	40	80	60
D-temperature (°C)	160	200	180
E-time (min)	30	90	60

Sample name	Samples and experimental conditions					Responses
	[H_2_SO_4_] step 1 (% v/v)	[Catalyst] step 2 (mol/L)	[Ethanol] step 2 (% v/v)	Temp step 2 (°C)	Time step 2 (min)	Reducing sugar release (mg/g)
EO1	None	None	40	160	90	58.5
EO2	1	None	40	160	30	95.1
EO3	None	0.06	40	160	30	98.0
EO4	1	0.06	40	160	90	102.9
EO5	None	None	80	160	30	43.6
EO6	1	None	80	160	90	91.7
EO7	None	0.06	80	160	90	143.6
EO8	1	0.06	80	160	30	120.6
EO9	None	None	40	200	30	77.3
EO10	1	None	40	200	90	82.2
EO11	None	0.06	40	200	90	59.2
EO12	1	0.06	40	200	30	56.1
EO13	None	None	80	200	90	90.3
EO14	1	None	80	200	30	73.7
EO15	None	0.06	80	200	30	109.9
EO16	1	0.06	80	200	90	5.0
EO17	0.5	0.03	60	180	60	100.4
EO18	0.5	0.03	60	180	60	106.1
EO19	0.5	0.03	60	180	60	95.6

The first acid step was carried out exactly as described previously, using H_2_SO_4_ solutions at 0.5 or 1% (v/v), or no acid step, as indicated in Table 3. Rinsed and dried samples after the acid pretreatment (or dried in natura samples when no acid step was applied) were placed in stainless steel reactors (total volume = 200 mL), together with the catalyst and the ethanol in distilled water mixture, keeping a 1:10 solid to total liquid ratio. The moisture content was also considered in this calculation. Ethanol and catalyst were added in different concentrations for each sample, as shown in Table 3. The reactors were heated in a silicone oil bath (at 160, 180 or 200 °C) for 30, 60 or 90 min, and then immersed in an ice bath to cool quickly at room temperature (ca. 3 min). Solids were separated from the hydrolysate by filtration in cotton tissue, rinsed with 100 mL ethanol first and then with tap water and, finally, oven dried at 60 °C for 7 h.

#### Experimental design analysis

The experimental variables studied in the $2_{\text{V}}^{{ 5 { - 1}}}$ fractional factorial design for each sample are specified in Tables 2 and 3. Central points had two main objectives: (1) provide an additional level for lack of fit testing (if all coefficients were significant) and (2) provide degrees of freedom for pure error estimation, due to the replication of experiments at this point. The half-normal plot of the effects was used to select the significant factors that influence sugar release [19, 20]. The significant coefficients were selected to be included in a model, in addition to coefficients required to keep the hierarchy of the model.

Analysis of variance (ANOVA) was used to test the regression significance and model lack of fit by means of *F* tests. Regression was considered significant if the regression mean square (MS_REG_) was statistically greater than the residual mean squares (MS_RES_), indicating that the variation in the dependent variable was indeed caused by the variations of the independent variables. The model was considered to present a good fit to the experimental data if the lack of fit mean square (MS_LOF_) was equivalent to pure error mean square (MS_PE_) [19, 20]. Graphs of residuals and predicted vs actual values were used as auxiliary diagnostics tools. Response surfaces were built to describe the behaviour of the response over the experimental domain and select the conditions that lead to the maximization of sugar release. Design Expert software (StatEase, Minneapolis) v 9.0.6 was used to build the design and analyse the data.

#### Sample characterization

Samples were ground to a fine powder in a ball mill (TissueLyser II, Qiagen (Hilden, Germany) for 30 s at 30 Hz prior to compositional analysis.

#### Analysis of matrix polysaccharides

Ground biomass samples (4 mg) contained in 2 mL tubes were hydrolysed in 0.5 mL 2 mol/L trifluoroacetic acid (TFA) solution for 4 h at 100 °C in argon atmosphere. Solids were then rinsed twice with 0.5 mL 2-propanol, evaporated in a speed-vac concentrator (Savant SPD131DDA, Thermo Scientific) and resuspended in 0.2 mL MilliQ water under vigorous agitation. This suspension was centrifuged at 1500 rpm for 5 min and the supernatant was collected for soluble monosaccharide analysis (hemicellulose fraction). Samples were filtered with 0.45 µm polytetrafluoroethylene (PTFE) filters and separated by high-performance anion exchange chromatography (HPAEC), using a Dionex Carbopac PA-10 column, as described in Jones et al. [26]. The separated monosaccharides were quantified using an external calibration containing seven monosaccharide standards at 100 µmol/L (arabinose, fucose, galactose, glucose, mannose, rhamnose, and xylose) that were subjected to acid hydrolysis in parallel with the samples.

The remaining hemicellulose fraction in the samples was calculated considering the quantification of hemicellulose sugars in the solids after pretreatments (Figs. 2a, 3a) and the hydrolysed fraction (weight loss) in each pretreatment step (total solid remaining in Additional file 1: Figure S9). The initial amounts of the components in EIN were considered as 100% to calculate the remaining percentages in the pretreated samples.

The residual solid pellets were used to determine the total cellulose content in each sample [27]. After TFA hydrolysis and supernatant collection for analysis of hemicellulose fractions, the solids were first rinsed once with 1.5 mL distilled water, by vortexing, centrifuging and discarding the supernatant, and then rinsed three times with 1.5 mL acetone, following the same steps. After drying under evaporation overnight, the pellets were hydrolysed with 90 µL 72% H_2_SO_4_ (w/w) for 4 h at room temperature. Acid concentration was then diluted to 3.2% by adding distilled water and the hydrolysis continued for another 4 h at 120 °C. After cooling to room temperature, samples were centrifuged at 1500 rpm for 10 min and the cellulose content was determined by the colorimetric anthrone method [28]. Matrix polysaccharides were determined in duplicate.

#### Acetyl bromide soluble lignin quantification

Total lignin was determined in solid samples before and after pretreatments following the colorimetric method based on lignin dissolution in acetyl bromide [29]. Ground samples (4 mg) were hydrolysed with 250 µL of a freshly prepared 25% v/v acetyl bromide in acetic acid solution in 2 mL tubes. Samples were kept at 50 °C for 3 h under periodic stirring. After cooling to room temperature, the hydrolysed sample was transferred to a 5 mL volumetric flask and the tube was rinsed with 1 mL of a 2 M NaOH solution that was also added to the flask. Next, 175 µL of a 0.5 mol/L solution of hydroxylamine in HCl was added to each sample, followed by vigorous vortexing. Finally, the volume was completed to 5 mL with glacial acetic acid, the solution was diluted 1:10 and the absorbance was measured at 280 nm in a spectrophotometer. Soluble lignin concentration was determined in duplicate using the absorption coefficient for grasses (17.75 L/g cm).

#### Silica quantification by X-ray fluorescence spectroscopy (XRF)

Silicon was determined following a procedure previously reported and validated [30], using an X-ray fluorescence spectrometer (Niton XL3t900 GOLDD Analyser, Thermo Scientific, Winchester, UK) equipped with an X-ray tube and a silicon drift detector. All measurements were carried out in duplicate in dried and ground biomass samples pressed into pellet form.

#### Determination of total solids and ash content

The moisture contents (or total solids) of the samples (1 g) were determined in triplicate, using a heating balance (Metler Toledo, Switzerland). Ash contents were determined in duplicate by total calcination of 1 g of solid biomass samples in muffle oven (EDG F-1800 10P, São Carlos, Brazil) at 600 °C for 24 h.

#### Automated enzymatic saccharification

Automated saccharification assays were performed based on Gomez et al. [31]. Hydrolysis was carried out in a monitored shaking incubator (Tecan Group Ltd.) using an enzyme cocktail with a 4:1 ratio of Celluclast and Novozyme 188 (both from Novozymes, Bagsvaerd, Denmark) in a minimum of 4 replicates. Hydrolysis conditions were 50 °C, pH 4.5 (25 mmol/L sodium acetate buffer) for 12 h, with enzyme loading of 8 FPU/g biomass and total liquid volume of 850 µL. Prior to incubation, biomass substrates underwent a 2 h hydration step in the buffer at room temperature. Automated determination of released reducing sugar after hydrolysis was performed using 3-methyl-2-benzothiazolinone hydrazone, as previously described [31, 32].

## Additional file

**Additional file 1: Figure S1.** Sugar release as a function of lignin content in samples EA1–19 (*R* = − 0.67). **Figure S2**. Sugar release as a function of cellulose content in samples EA1–19 (*R* = 0.41). **Figure S3**. Sugar release as a function of silica content in samples EA1–19 (*R* = − 0.71). **Figure S4**. Sugar release as a function of lignin content in samples EO1–19 (*R* = − 0.67). **Figure S5**. Sugar release as a function of cellulose content in samples EO1–19 (*R* = 0.80). **Figure S6**. Sugar release as a function of silica content in samples EO1–19 (*R* = 0.20). **Figure S7**. Comparison of silica and total ash amounts before and after pretreatments in (**a**) EA, and (**b**) EO. **Figure S8**. Crystallinity index (%) obtained by x-ray diffraction (XRD) for elephant grass samples in natura after the corresponding ball milling times. **Figure S9**. Remaining fraction of solids (considering the initial amount in sample in natura) in elephant grass samples before (EIN) and after the acid-alkali pretreatments: (**a**) EA and (**b**) EO. **Figure S10**. (**a**) Internally studentized residuals (residuals/standard deviation of the regression) vs predicted values of reducing sugars as provided by the selected model obtained for EO samples and (**b**) Predicted values vs actual experimental values of reducing sugars. **Table S1**. Levels of the factors evaluated in the 2V5-1 fractional factorial design, sample identification with the corresponding experimental conditions and two responses evaluated in the acid-alkali pretreatment applied to elephant grass leaves (EA). **Table S2**. ANOVA table of the model describing the lignin amount as a linear function of the selected coefficients for EA samples, as obtained from Design Expert software. Significant factors are highlighted. **Table S3**. ANOVA table of the model describing the silica amount as a linear function of the selected coefficients for EA samples, as obtained from Design Expert software. Significant factors are highlighted. **Table S4**. Levels of the factors evaluated in the 2V5-1 fractional factorial design, sample identification with the corresponding experimental conditions and two responses evaluated in the organosolv pretreatment applied to elephant grass leaves (EO). **Table S5**. ANOVA table of the model describing the lignin amount as a linear function of the selected coefficients for EO samples, as obtained from Design Expert software. The significant factor is highlighted. **Table S6**. ANOVA table of the model describing the cellulose amount as a linear function of the selected coefficients for EO samples, as obtained from Design Expert software. Significant factors are highlighted.

## Abbreviations

DOE: design of experiments
EA: elephant grass pretreated with acid–alkali
EH: elephant grass pretreated with acid only
EIN: elephant grass in natura
EO: elephant grass pretreated with acid–organosolv
FFD: fractional factorial design
HPAEC: high performance anion exchange chromatography
MLR: multiple linear regression
MS_LOF_: lack of fit mean square
MS_PE_: pure error mean square
MS_REG_: regression mean square
MS_RES_: residual mean square
XRD: X-ray diffraction
XRF: X-ray fluorescence spectroscopy
