# Supplementary material for: Optimization of biomass pretreatments using fractional factorial experimental design
Source: Biotechnol Biofuels. 2018 Jul 24;11:206. doi: 10.1186/s13068-018-1200-2 (PMC6058377; doi:10.1186/s13068-018-1200-2)
Supplement: Supplementary file 1 — Additional file 1: Figure S1. Sugar release as a function of lignin content in samples EA1–19 (R = − 0.67). Figure S2. Sugar release as a function of cellulose content in samples EA1–19 (R = 0.41). Figure S3. Sugar release as a function of silica content in samples EA1–19 (R = − 0.71). Figure S4. Sugar release as a function of lignin content in samples EO1–19 (R = − 0.67). Figure S5. Sugar release as a function of cellulose content in samples EO1–19 (R = 0.80). Figure S6. Sugar release as a function of silica content in samples EO1–19 (R = 0.20). Figure S7. Comparison of silica and total ash amounts before and after pretreatments in (a) EA, and (b) EO. Figure S8. Crystallinity index (%) obtained by x-ray diffraction (XRD) for elephant grass samples in natura after the corresponding ball milling times. Figure S9. Remaining fraction of solids (considering the initial amount in sample in natura) in elephant grass samples before (EIN) and after the acid-alkali pretreatments: (a) EA and (b) EO. Figure S10. (a) Internally studentized residuals (residuals/standard deviation of the regression) vs predicted values of reducing sugars as provided by the selected model obtained for EO samples and (b) Predicted values vs actual experimental values of reducing sugars. Table S1. Levels of the factors evaluated in the 2V5-1 fractional factorial design, sample identification with the corresponding experimental conditions and two responses evaluated in the acid-alkali pretreatment applied to elephant grass leaves (EA). Table S2. ANOVA table of the model describing the lignin amount as a linear function of the selected coefficients for EA samples, as obtained from Design Expert software. Significant factors are highlighted. Table S3. ANOVA table of the model describing the silica amount as a linear function of the selected coefficients for EA samples, as obtained from Design Expert software. Significant factors are highlighted. Table S4. Levels of the factors evaluated in the 2V5-1 fr [file 13068_2018_1200_MOESM1_ESM.docx]

**Supplementary material for the paper**

**Optimization of biomass pretreatments using fractional factorial experimental design**

Camila A. Rezende*^1^; Beatriz W. Atta^1^, Marcia C. Breitkreitz^1^, Rachael Simister^2^, Leonardo D. Gomez^2^; Simon J. McQueen-Mason^2^

^1^ Institute of Chemistry, University of Campinas - UNICAMP, P.O. Box 6154, 13083-970, Campinas, SP, Brazil.

^2^ Centre for Novel Agricultural Products-CNAP, University of York, Heslington – York YO10 5YW – UK.

^*^ Corresponding author: [camila@iqm.unicamp.br](mailto:camila@iqm.unicamp.br), Phone: +55-19-35212104

1. **Correlation between saccharification results (reducing sugar release – mg/g) and lignin, cellulose and silica contents (w/w%) in EA and EO samples.**

Figures S1 to S3 show the correlation between reducing sugar release and lignin, cellulose and silica, respectively, for elephant grass samples that underwent the acid-alkali pretreatment (EA). A linear curve was fitted to the data presented in every plot (curve in red) and a correlation coefficient (Pearson’s R) was calculated.

In EA, the correlation between sugar release and lignin or silica can be considered relatively high (R=-0.67 for lignin and R=-0.71 for silica). Both values are negative, indicating that higher sugar release is associated with low amounts of lignin and silica (Figures S1 and S3). On the other hand, the correlation between sugar release and cellulose amounts in EA samples is positive but low (R=0.41 in Figure S2).


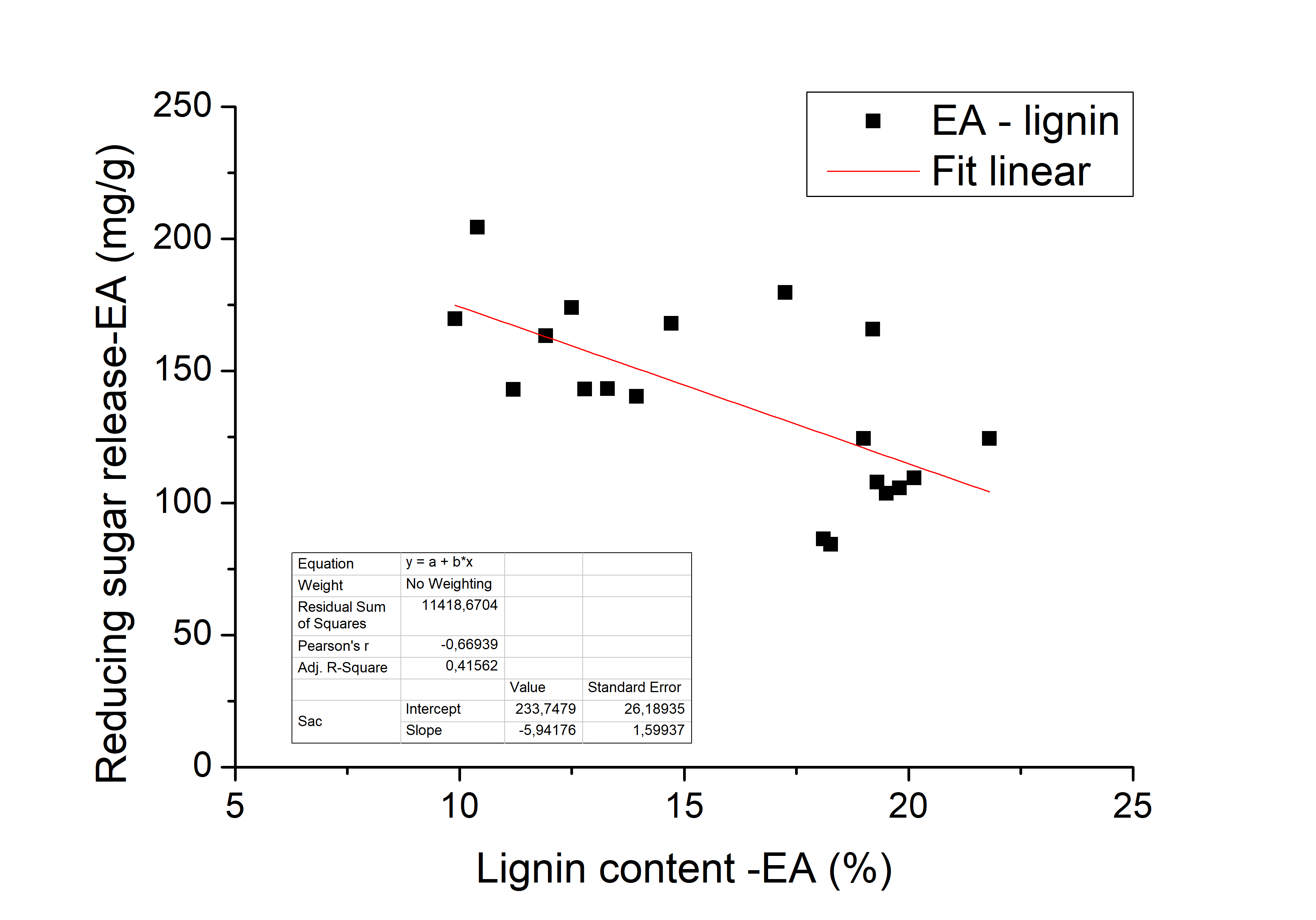


**Figure S1.** Sugar release as a function of lignin content in samples EA1-19 (R=-0.67).

**
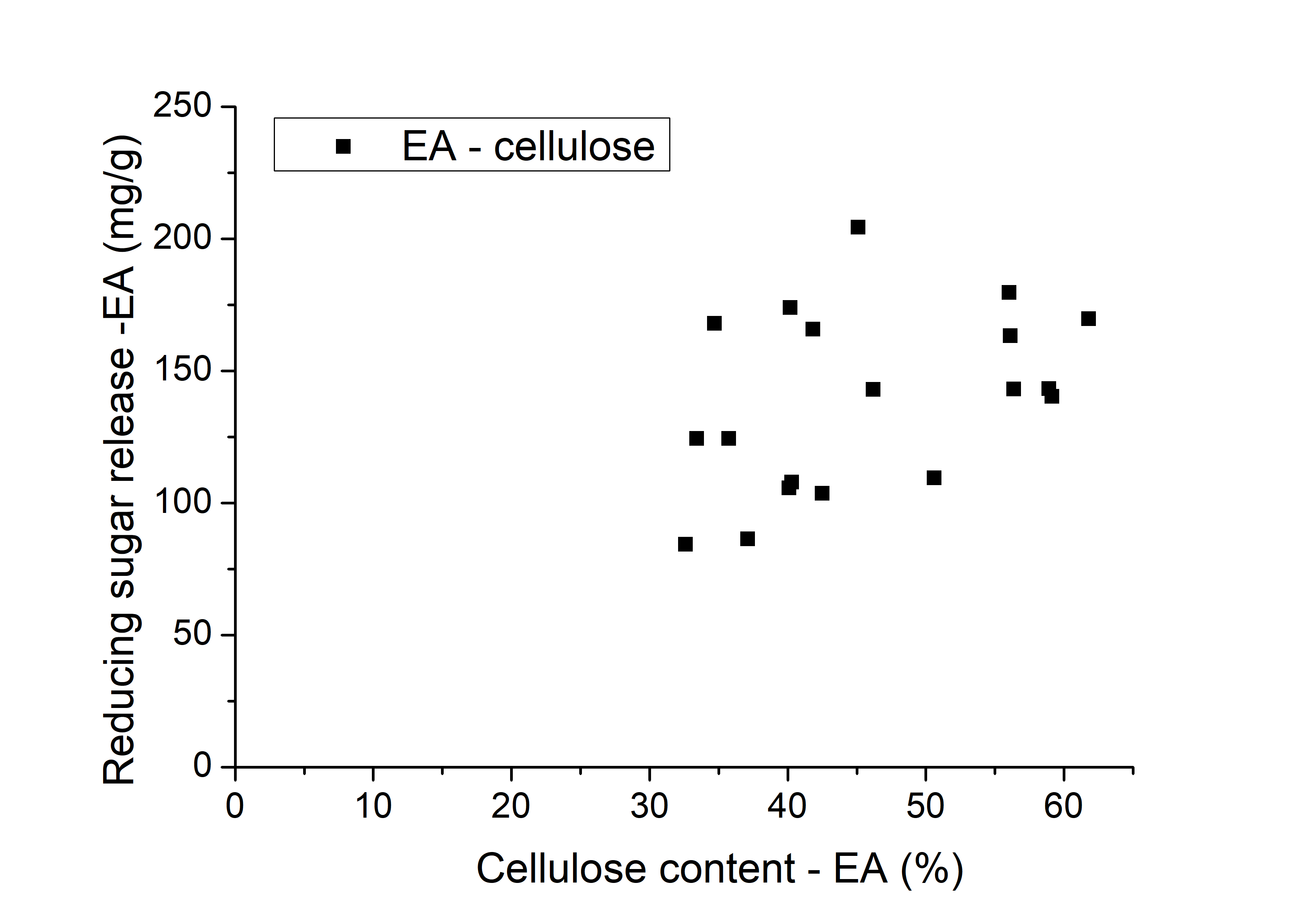
**

**Figure S2.** Sugar release as a function of cellulose content in samples EA1-19 (R=0.41).


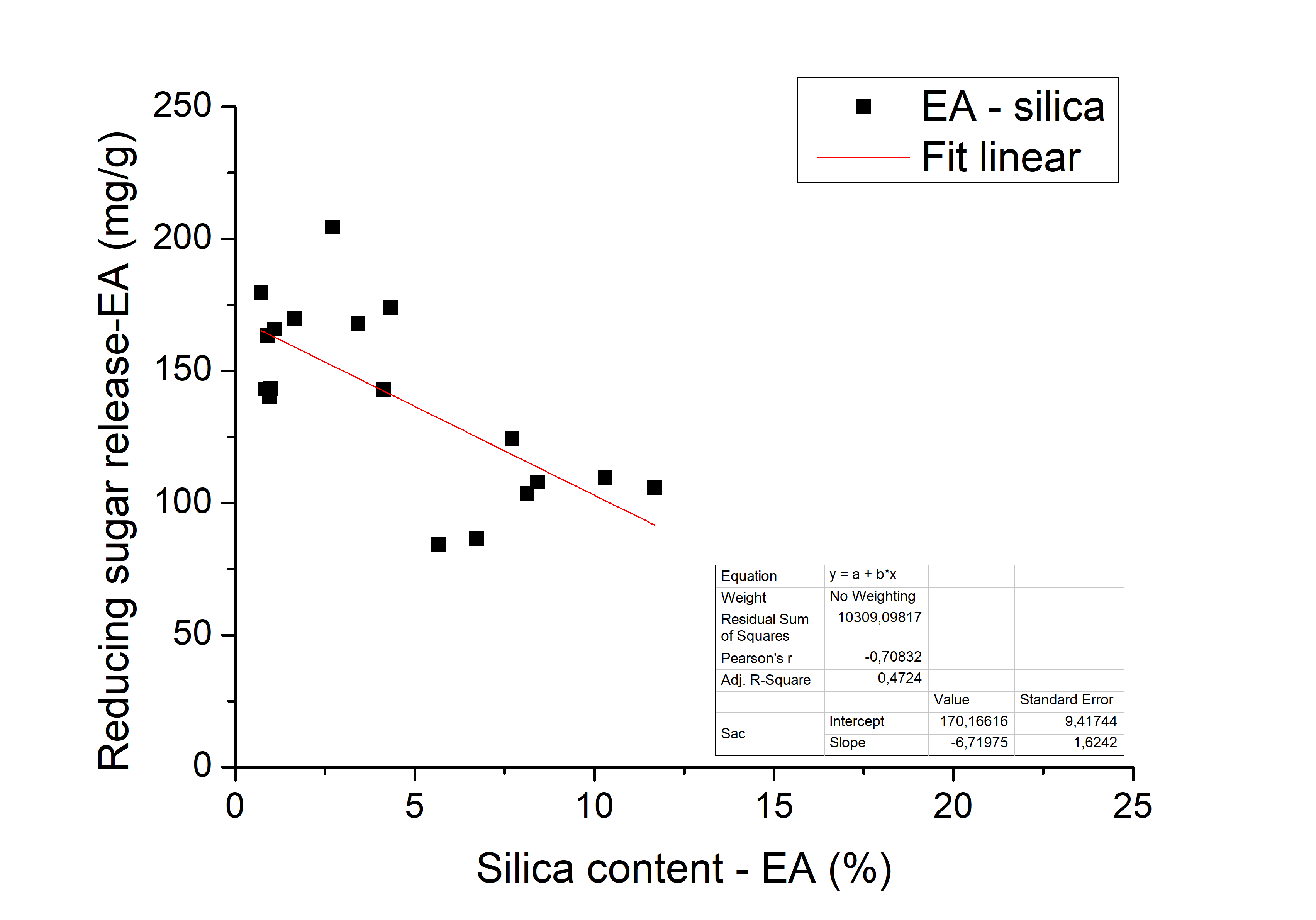


**Figure S3.** Sugar release as a function of silica content in samples EA1-19 (R=-0.71).

Figures S4 to S6 show the correlation between reducing sugar release and lignin, cellulose and silica, respectively, for elephant grass samples that underwent the acid-organosolv pretreatment (EO). In these samples, the correlation between sugars and lignin (R=-0.67 in Figure S4) is the same as observed in EA samples. The correlation between sugar release and cellulose is positive and relatively high (R=0.80), indicating that higher sugar release is achieved in samples rich in cellulose (Figure S5). In terms of silica amounts in EO samples, a poor correlation is observed between silica and the sugar released (R=0.20).


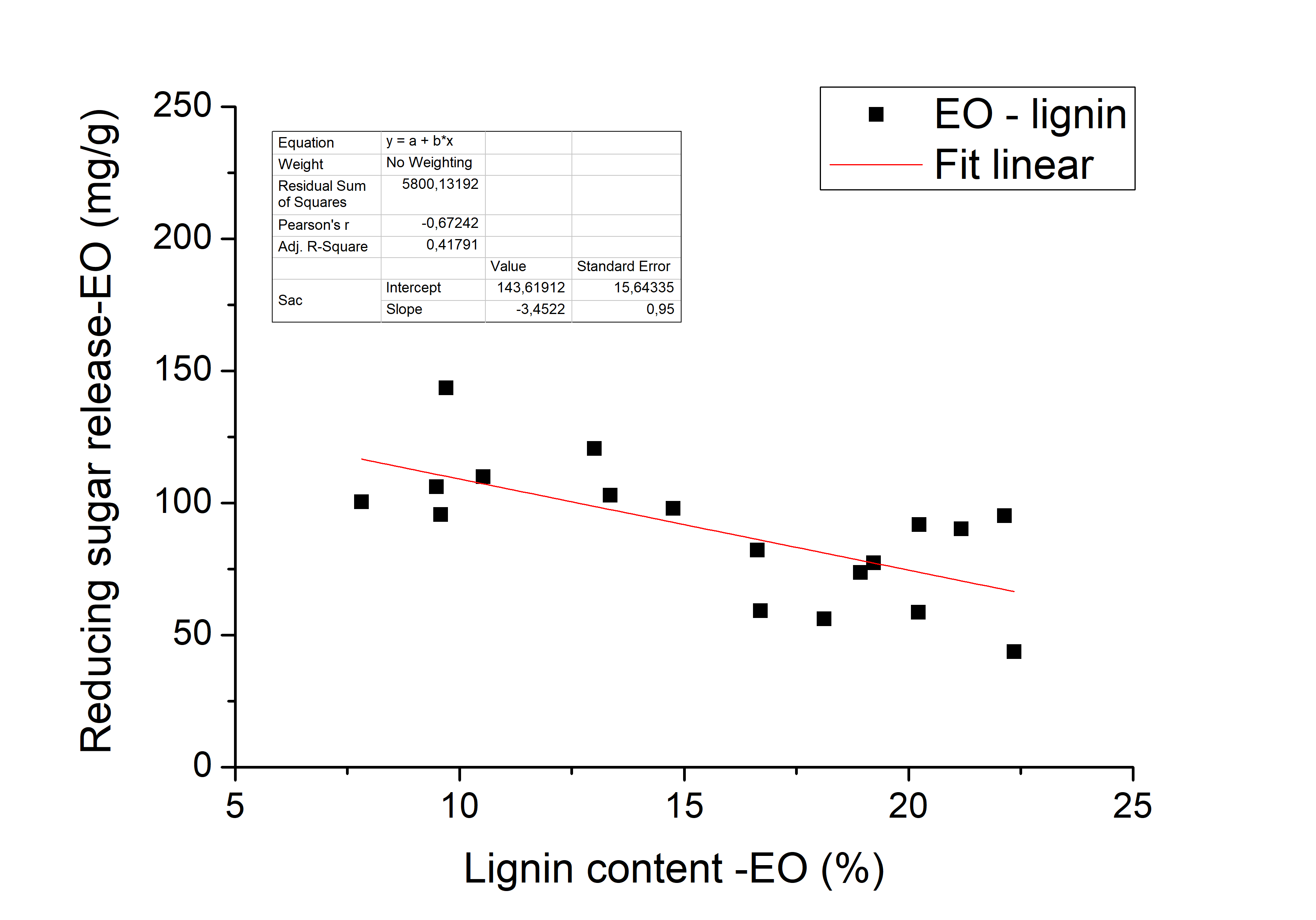


**Figure S4.** Sugar release as a function of lignin content in samples EO1-19 (R=-0.67).


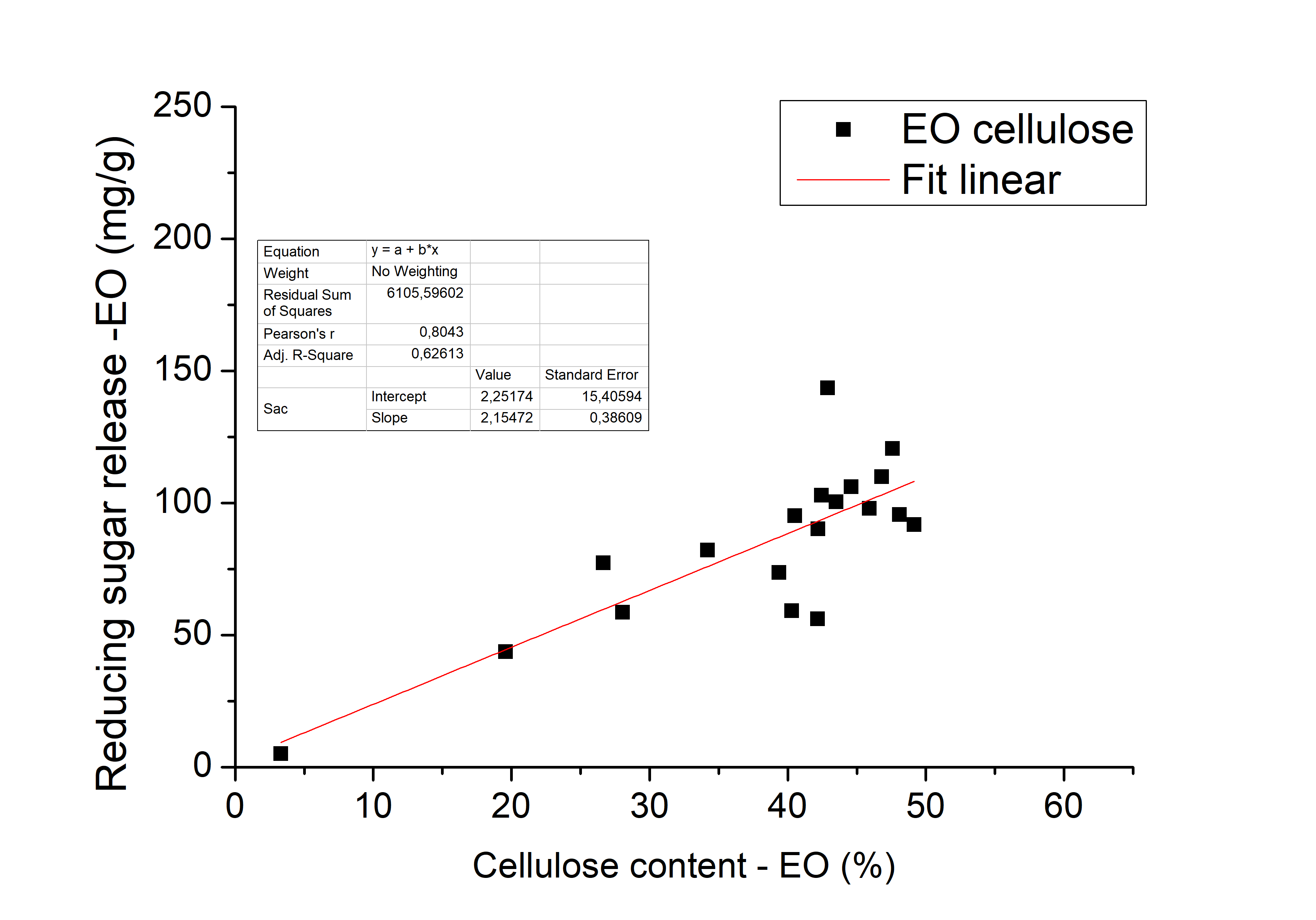


**Figure S5.** Sugar release as a function of cellulose content in samples EO1-19 (R=0.80).


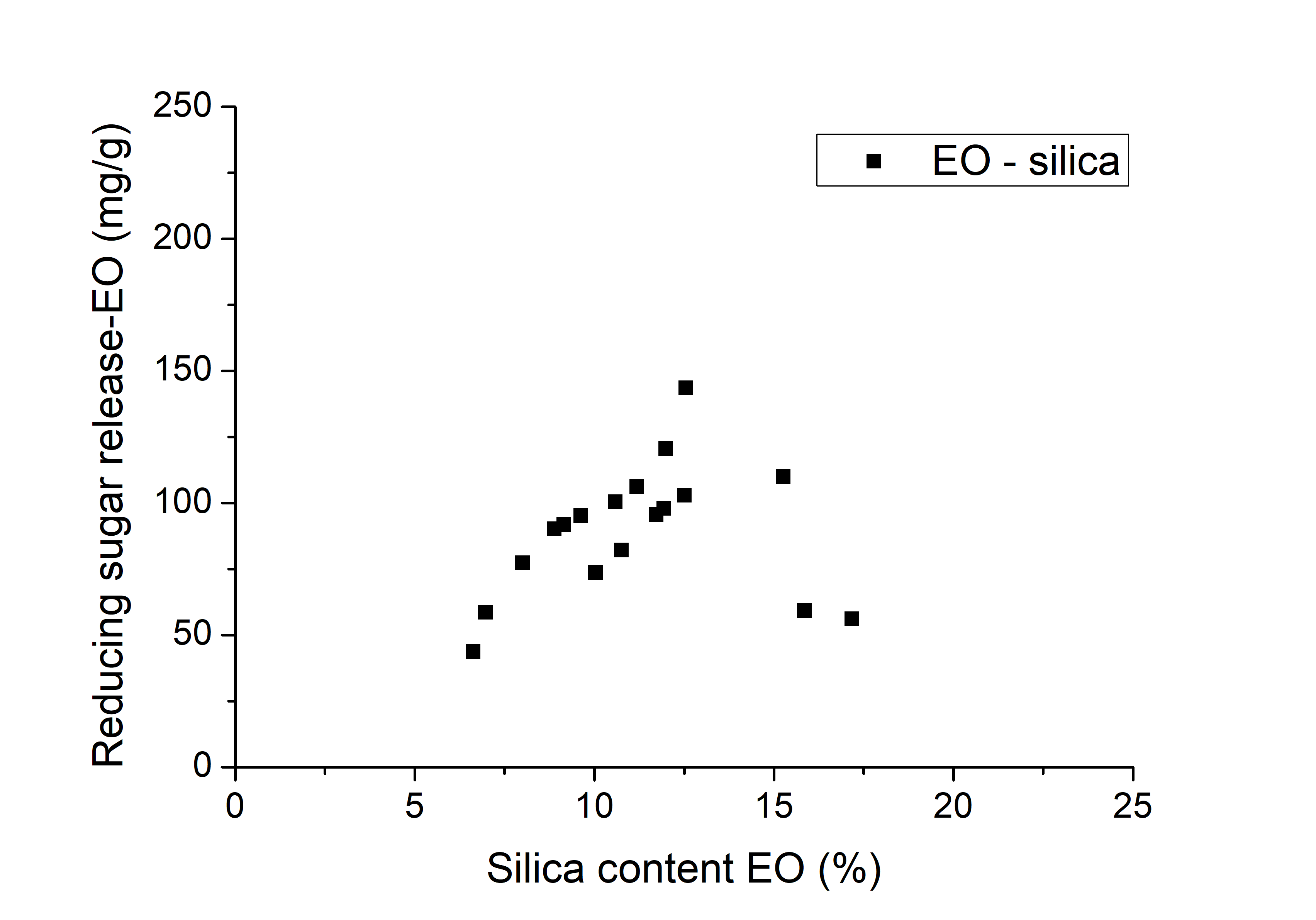


**Figure S6.** Sugar release as a function of silica content in samples EO1-19 (R=0.20).

1. **Quantification of silica and total ashes**


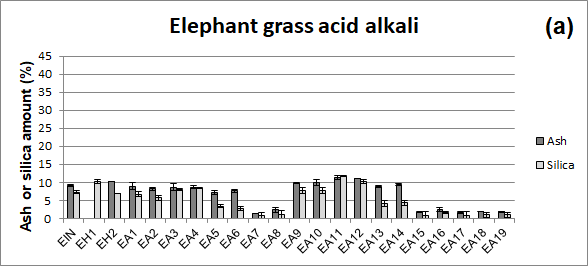


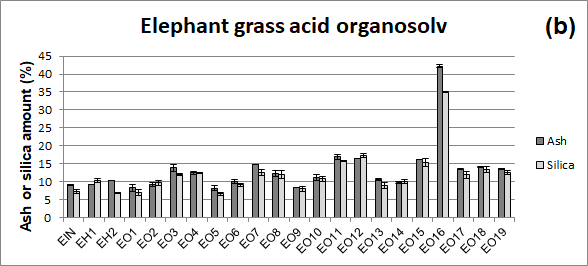


**Figure S7.** Comparison of silica and total ash amounts before and after pretreatments in (a) EA, and (b) EO.

1. **Crystallinity index of elephant grass samples *in natura*, as determined by x-ray diffraction after specific ball milling times**

Elephant grass samples after different ball milling times were collected to have their crystallinity evaluated by x-ray diffraction (XRD). XRD analyses were performed using a Shimadzu diffractometer model XRD 7000 (Cu target, Kα radiation with λ = 0.15406 nm, operating at 40kV and 30mA). Measurements were performed at room temperature at a 2θ angular range from 5 to 60° at a scan rate of 2°θ/min. The method proposed by Segal *et. al.* [1] ^[[1]](#footnote-1)^was used to calculate the crystallinity index (IC) of the solids. Figure S8 shows that the crystallinity indexes calculated for elephant grass samples *in natura* (IC=59% initially) decreased to *ca.* 50% as the ball milling time increased up to 10h. Ball milling for periods longer than 10h did not result in further decrease in CI values in these samples, using this mill.


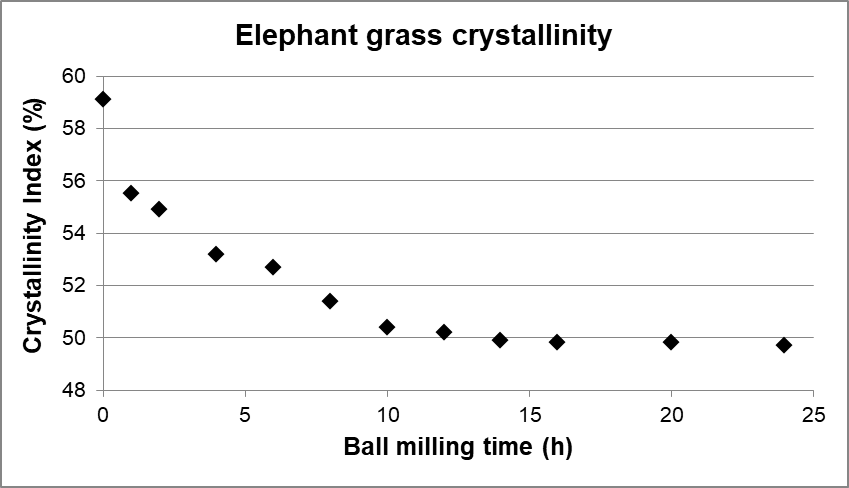


**Figure S8.** Crystallinity index (%) obtained by x-ray diffraction (XRD) for elephant grass samples *in natura* after the corresponding ball milling times.

1. **Total solid remaining in the samples**

Figure S9 shows the percentage of total solids remaining in the biomass after the different pretreatments (EA and EO), calculated considering the initial dry weight of biomass *in natura* (EIN). In EA and EO, it is possible to observe different amounts of remaining solids for different pretreatment conditions. The most severe pretreatments in EA are EA3, EA15, and EA16, followed by the samples of the central point. On the other hand, the less severe pretreatments (the ones where the amount of remaining solids are high) are EA1, EA2, EA9, EA11 and EH1 and EH2. All of them contain only one step, except EA11.

In EO, the less severe pretreatments were EO1, EO5, EO9 and EO13, which are the ones that use no catalyst and no acid step. The most severe pretreatments in EO, on the other hand, were EO11, EO12, EO16 and the central points. EO16 is the pretreatment that have all the factors at high levels, while EO11 and EO12 both use catalyst (0.06M) and temperature at high level (200°C).


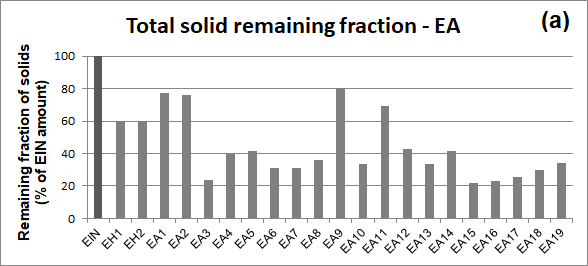


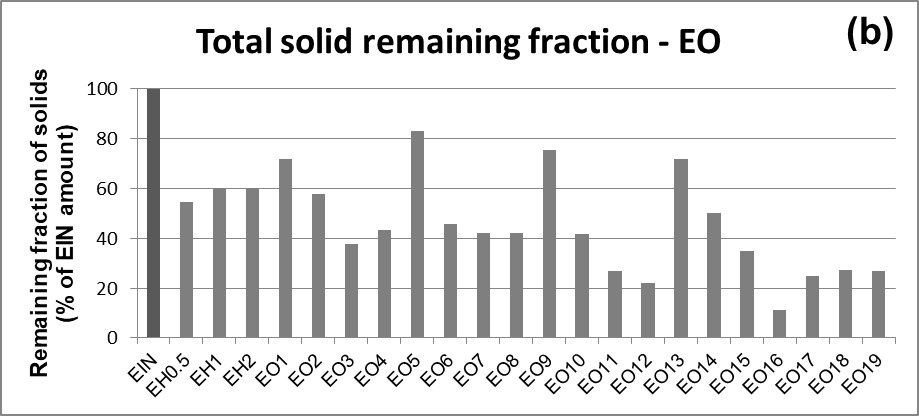


**Figure S9.** Remaining fraction of solids (considering the initial amount in sample *in natura*) in elephant grass samples before (EIN) and after the acid-alkali pretreatments: (a) EA and (b) EO.

1. **Supplementary data obtained in the experimental design analysis for EO samples.**

The diagnostics graphs of residuals *vs* predicted values as provided by the model obtained for EO samples is presented in Figure S10a and indicates a random distribution of the residuals without heteroscedasticity. The graph of predicted *vs* actual experimental responses (Figure S10b) indicates that the linear model describes very well the experimental behavior.


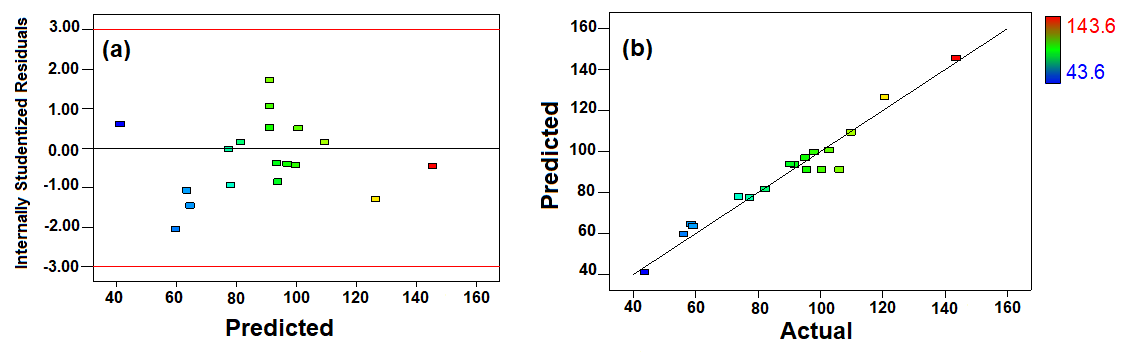


**Figure S10.** **(a)** Internally studentized residuals (residuals/standard deviation of the regression) *vs* predicted values of reducing sugars as provided by the selected model obtained for EO samples and **(b)** Predicted values *vs* actual experimental values of reducing sugars.

1. **ANOVA for responses other than sugar release**

**Lignin and silica amounts as responses in EA samples**

Pretreatment conditions were determined following a 2_V_^5-1^ fractional factorial design, with triplicates in the central point to evaluate reducing sugars, lignin and silica percentages in the solid samples as responses. Table S1 shows the levels within which the effects are varied and the corresponding sample name.

Table S2 shows the analysis of variance (ANOVA) of the model describing the lignin amount as a linear function of the selected coefficients for EA samples. The significant factors are the ones with *p-value* <0.05, named NaOH concentration in step 2 and the interaction between NaOH concentration and temperature (CD). ANOVA indicated a low significant regression, with lack of fit of the linear model, but this does not invalidate the significance of the factors.

**Table S1.** Levels of the factors evaluated in the 2_V_^5-1^ fractional factorial design, sample identification with the corresponding experimental conditions and two responses evaluated in the acid-alkali pretreatment applied to elephant grass leaves (EA).

| **Factor levels** | | | | | | | |
| --- | --- | --- | --- | --- | --- | --- | --- |
|  | | **Low level (-)** | | **High level (+)** | | **Central (0)** | |
| **A- Ball mill time (h)** | | 0 | | 10 | | 5 | |
| **B- [H_2_SO_4_] (%v/v)** | | none | | 2 | | 1 | |
| **C- [NaOH] (%w/v)** | | 0.5 | | 4.5 | | 2.5 | |
| **D- Temperature (°C)** | | 85 | | 125 | | 105 | |
| **E- Time (min)** | | 20 | | 80 | | 60 | |
| **Samples and experimental conditions** | | | | | | **Responses** | |
| **Sample name** | Ball mill time  (h) | [H_2_SO_4_] step 1 (% v/v) | [NaOH] step 2 (% m/v) | Temp step 2  (°C) | Time step 2 (min) | Lignin amount (%) | Silica amount (%) |
| **EA1** | 0 | none | 0.5 | 85 | 100 | 18.1 | 6.72 |
| **EA2** | 10 | none | 0.5 | 85 | 20 | 18.3 | 5.67 |
| **EA3** | 0 | 2 | 0.5 | 85 | 20 | 19.5 | 8.13 |
| **EA4** | 10 | 2 | 0.5 | 85 | 100 | 19.3 | 8.42 |
| **EA5** | 0 | none | 4.5 | 85 | 20 | 14.7 | 3.42 |
| **EA6** | 10 | none | 4.5 | 85 | 100 | 10.4 | 2.72 |
| **EA7** | 0 | 2 | 4.5 | 85 | 100 | 17.3 | 0.73 |
| **EA8** | 10 | 2 | 4.5 | 85 | 20 | 19.2 | 1.09 |
| **EA9** | 0 | none | 0.5 | 125 | 20 | 21.8 | 7.71 |
| **EA10** | 10 | none | 0.5 | 125 | 100 | 19.0 | 7.71 |
| **EA11** | 0 | 2 | 0.5 | 125 | 100 | 19.8 | 11.7 |
| **EA12** | 10 | 2 | 0.5 | 125 | 20 | 20.1 | 10.3 |
| **EA13** | 0 | none | 4.5 | 125 | 100 | 11.2 | 4.14 |
| **EA14** | 10 | none | 4.5 | 125 | 20 | 12.5 | 4.33 |
| **EA15** | 0 | 2 | 4.5 | 125 | 20 | 11.9 | 0.90 |
| **EA16** | 10 | 2 | 4.5 | 125 | 100 | 9.9 | 1.65 |
| **EA17** | 5 | 1 | 2.5 | 105 | 60 | 12.8 | 0.86 |
| **EA18** | 5 | 1 | 2.5 | 105 | 60 | 13.9 | 0.96 |
| **EA19** | 5 | 1 | 2.5 | 105 | 60 | 13.3 | 0.98 |

**Table S2.** ANOVA table of the model describing the lignin amount as a linear function of the selected coefficients for EA samples, as obtained from Design Expert software. Significant factors are highlighted.


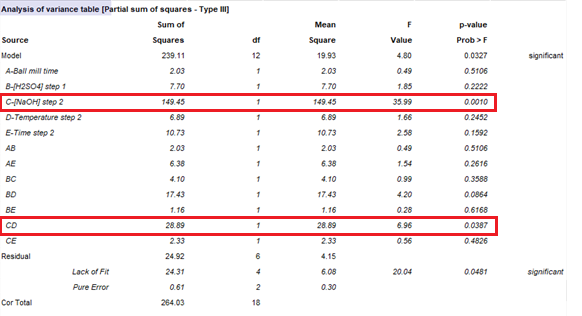


Table S3 shows ANOVA of the model describing the silica amount as a linear function of the selected coefficients for EA samples. The significant factors, in this case, are NaOH concentration in step 2 and its interaction with H_2_SO_4_ concentration in step 1, as indicated in red. Again, the lack of fit is significant, but it does not invalidate the calculation of the effects.

**Table S3.** ANOVA table of the model describing the silica amount as a linear function of the selected coefficients for EA samples, as obtained from Design Expert software. Significant factors are highlighted.


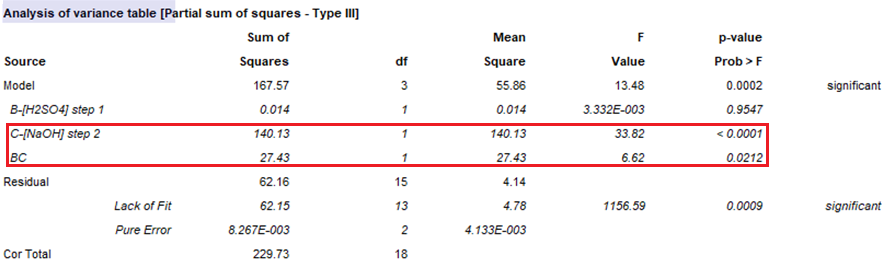


**Lignin and cellulose amounts as responses in EO samples**

Table S4 shows the levels of the factors and the responses (lignin and cellulose percentages in the solid samples) evaluated in the acid-organosolv pretreatment. Table S5 shows ANOVA of the model describing the lignin amount as a linear function of the selected coefficients for EO samples. The only significant factor (*p-value* <0.05) for this response is the catalyst concentration in step 2, as highlighted.

In Table S6, ANOVA of the model describing the cellulose content amount as a linear function of the selected coefficients for EO samples is presented. The significant factors here are the concentration of H_2_SO_4_ in step 1, the catalyst in step 2 and the ethanol in step 2, as highlighted. Also, the interaction between ethanol concentration and time (CE) presents a *p-value*<0.05 (statistically significant). The predictive capability of the model is also very poor here, despite the regression and lack of fit values presented in Table S4. The incorrect assignments as significant or insignificant for regression and lack of fit, respectively, are a consequence of the very small degrees of freedom in this system.

**Table S4.** Levels of the factors evaluated in the 2_V_^5-1^ fractional factorial design, sample identification with the corresponding experimental conditions and two responses evaluated in the organosolv pretreatment applied to elephant grass leaves (EO).

| **Factor levels** | | | | | | | | | |
| --- | --- | --- | --- | --- | --- | --- | --- | --- | --- |
|  | | **Low level (-)** | | **High level (+)** | | | | **Central (0)** | |
| **A- [H_2_SO_4_] (%v/v)** | | none | | 1 | | | | 0.5 | |
| **B- [catalyst] (mol/L)** | | 0 | | 0.06 | | | | 0.03 | |
| **C- [ethanol] (%v/v)** | | 40 | | 80 | | | | 60 | |
| **D- Temperature (°C)** | | 160 | | 200 | | | | 180 | |
| **E- Time (min)** | | 30 | | 90 | | | | 60 | |
|  | **Samples and experimental conditions** | | | | | | | **Responses** | |
| Sample name | [H_2_SO_4_] step 1 (% v/v) | [catalyst] step 2  (mol/L) | [ethanol] step 2 (% v/v) | | Temp step 2  (°C) | Time step 2 (min) | Lignin amount (%) | | Cellulose amount (%) |
| **EO1** | none | none | 40 | | 160 | 90 | 20.2 | | 28.0 |
| **EO2** | 1 | none | 40 | | 160 | 30 | 22.1 | | 40.5 |
| **EO3** | none | 0.06 | 40 | | 160 | 30 | 14.8 | | 45.9 |
| **EO4** | 1 | 0.06 | 40 | | 160 | 90 | 13.4 | | 42.5 |
| **EO5** | none | none | 80 | | 160 | 30 | 22.4 | | 19.6 |
| **EO6** | 1 | none | 80 | | 160 | 90 | 20.2 | | 49.1 |
| **EO7** | none | 0.06 | 80 | | 160 | 90 | 9.7 | | 42.9 |
| **EO8** | 1 | 0.06 | 80 | | 160 | 30 | 13.0 | | 47.6 |
| **EO9** | none | none | 40 | | 200 | 30 | 19.2 | | 26.6 |
| **EO10** | 1 | none | 40 | | 200 | 90 | 16.6 | | 34.2 |
| **EO11** | none | 0.06 | 40 | | 200 | 90 | 16.7 | | 40.3 |
| **EO12** | 1 | 0.06 | 40 | | 200 | 30 | 18.1 | | 42.2 |
| **EO13** | none | none | 80 | | 200 | 90 | 21.2 | | 42.2 |
| **EO14** | 1 | none | 80 | | 200 | 30 | 18.9 | | 39.4 |
| **EO15** | none | 0.06 | 80 | | 200 | 30 | 10.5 | | 46.8 |
| **EO16** | 1 | 0.06 | 80 | | 200 | 90 | --- | | 3.3 |
| **EO17** | 0.5 | 0.03 | 60 | | 180 | 60 | 7.8 | | 43.5 |
| **EO18** | 0.5 | 0.03 | 60 | | 180 | 60 | 9.5 | | 44.6 |
| **EO19** | 0.5 | 0.03 | 60 | | 180 | 60 | 9.6 | | 48.1 |

**Table S5.** ANOVA table of the model describing the lignin amount as a linear function of the selected coefficients for EO samples, as obtained from Design Expert software. The significant factor is highlighted.


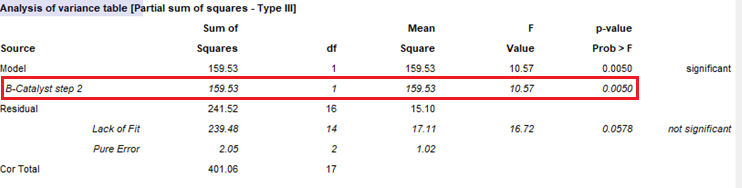


**Table S6.** ANOVA table of the model describing the cellulose amount as a linear function of the selected coefficients for EO samples, as obtained from Design Expert software. Significant factors are highlighted.


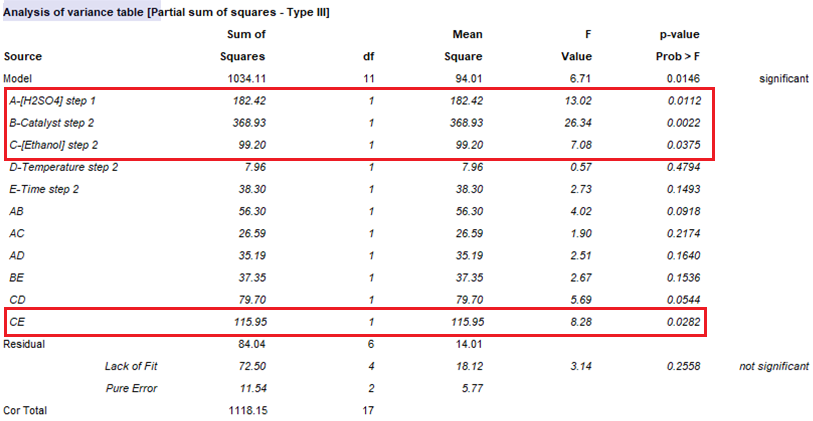


1. [1]Segal L, Creely JJ, Martin AE., Conrad CM. An empirical method for estimating the degree of crystallinity of native cellulose using the x-ray diffractometer. Text. Res. J. 1959;29:786–94. [↑](#footnote-ref-1)
